# Supplementary material for: Efficient and accurate frailty model approach for genome-wide survival association analysis in large-scale biobanks
Source: Nat Commun. 2022 Sep 16;13:5437. doi: 10.1038/s41467-022-32885-x (PMC9481565; doi:10.1038/s41467-022-32885-x)
Supplement: Supplementary file 1 — Supplementary Information [file 41467_2022_32885_MOESM1_ESM.pdf]

Efficient and accurate frailty model approach for genome-wide survival association analysis in large-scale biobanks – Supplementary Tables and Figures

Supplementary Tables

Supplementary Table 1: Projected computation time and memory usage for GATE, COXMEG-Score, and COXMEG-Sparse across different sample sizes. Benchmarking was performed for the genome-wide association study (GWAS) of lifespan based on randomly subsampled data from UK Biobank White British ancestry subjects. Association tests were performed on 200,000 randomly selected markers with imputation INFO ≥ 0.3, with the filtering criteria of minor allele count (MAC) ≥ 20. The computation times were projected for testing 46 million variants with INFO ≥ 0.3 and MAC ≥ 20. The reported run times are medians (means in parenthesis) of five runs, each with randomly sampled subjects with different randomization seeds.

Sample Size (N)	GATE		COXMEG-Score		COXMEG-Sparse	
	Time (CPU-hr)	Max. Memory (GB)	Time (CPU-hr)	Max. Memory (GB)	Time (CPU-hr)	Max. Memory (GB)
5000	27 (29)	0.36	192 (187)	1.80	356 (358)	0.47
10000	31 (31)	0.54	737 (746)	7.99	747 (748)	1.55
20000	34 (36)	0.74	3356 (3323)	32.75	1412 (1414)	5.95
50000	59 (57)	1.44				
100000	86 (85)	2.91				
200000	147 (151)	5.55				
408582	318 (330)	10.60				

Supplementary Table 2: Top genome-wide significant variants ($\alpha = 5\times10^{-8}$) in different loci based on GATE for lifespan based on the FinnGen Study and the UK Biobank data. For any variant with $p < 5\times10^{-8}$, we extend upstream and downstream by 1Mb, then merge the overlapping regions together to define the locus and report the variant that has the smallest p-value in each locus. Genomic coordinates are based on NCBI Build 37/UCSC hg19.

**Phenotype**	**Chromosome: Position**	**rsID**	**Nearest Gene**	**Function**	**REF**	**ALT**	**UK Biobank**				**Finngen Study**				**Meta-analysis UK Biobank + FinnGen**	
							**Imputation INFO**	**Allele frequncy**	**Hazard Ratio (95% CI)**	**p-value**	**Imputation INFO**	**Allele frequency**	**Hazard Ratio (95% CI)**	**p-value**	**Hazard Ratio (95% CI)**	**p-value**
Lifespan<br><br>UK Biobank<br>No. of subjects=406596,<br>No. of events=16875,<br>No. of censored=389721,<br>censoring rate = 95.8%<br><br>FinnGen study<br>No. of subjects=218396,<br>No. of events=15152,<br>No. of censored=203244,<br>censoring rate = 93.1%	19: 45424514	rs157592*¶	*APOC1; APOC1P 1*	intergenic	A	C	0.95	0.187	1.08 (1.05, 1.12)	1.87E-08				Not found in FinnGen	NA	NA
	19:45411941	rs429358*	*APOE*	missense	T	C	1	0.156	1.07 (1.04, 1.10)	1.92E-05	1	0.183	1.13 (1.10, 1.17)	1.01E-14	1.1(1.07-1.12)	4.04E-17

* rs157592 and rs429358 are in LD with $r^2 = 0.7$ in UK Biobank, rs429358 has p-value 1.83×10^{-6} based on SAIGE in UK Biobank. ¶rs157592 has p-value 3.16×10^{-9} based on SAIGE in UK Biobank.

Supplementary Table 3: Empirical type I error rates of GATE and GATE with no saddlepoint association (SPA) based on 9.4x10^8 association tests in 100 simulated data sets with censoring rates 50%, 75% and 90%, respectively. Each data set contains 5,000 independent individuals and 500 families. Each family was simulated following the pedigree structure shown in Supplementary Figure 6. The variance component parameter τ = 0.1 and 0.25.

Variance component parameter τ		Alpha	Censoring Rate		
			**50%**	**75%**	**90%**
**0.1**	**SPA**	**1.00E-06**	1.21E-06	1.11E-06	1.03E-06
		**5.00E-08**	7.41E-08	5.81E-08	5.22E-08
	**no SPA**	**1.00E-06**	1.27E-05	2.07E-05	5.12E-05
		**5.00E-08**	4.79E-06	8.90E-06	2.81E-05
**0.25**	**SPA**	**1.00E-06**	1.21E-06	1.08E-06	9.72E-07
		**5.00E-08**	6.12E-08	5.10E-08	5.53E-08
	**no SPA**	**1.00E-06**	1.20E-05	1.91E-05	4.67E-05
		**5.00E-08**	4.38E-06	8.18E-06	2.50E-05

Supplementary Table 4: Empirical type I error rates of GATE and GATE with no saddlepoint association (SPA) based on 8.3x10^8 association tests in 100 simulated data sets with censoring rates 50%, 75% and 90%, respectively. Each data set contains 10,000 randomly selected individuals with white British ancestry from the UK Biobank. The variance component parameter τ = 0.25.

		**Censoring rate**		
	**Alpha**	**50%**	**75%**	**90%**
**SPA**	**1.00E-06**	1.20E-06	1.09E-06	8.94E-07
	**5.00E-08**	7.09E-08	5.65E-08	3.85E-08
**no SPA**	**1.00E-06**	1.12E-05	1.90E-05	6.55E-05
	**5.00E-08**	4.35E-06	8.38E-06	3.99E-05

Supplementary Figures

Supplementary Figure 1: Comparing association p-values from GATE versus COXMEG based on 5 million genetic variants in simulation data sets. A. Scatter plots of association p-values from GATE versus COXMEG. B. Quantile-quantile plots stratified by minor allele frequency (MAF) for GATE and COXMEG. For each censoring rate, 100 data sets were simulated, each has 10,000 samples (5,000 independent samples and 500 families, each with 10 family members as shown in Supplementary Figure 6). For each data set, 50,000 simulated genetic markers were tested. C. Scatter plots of association p-values from GATE-noSPA versus COXMEG.

A.

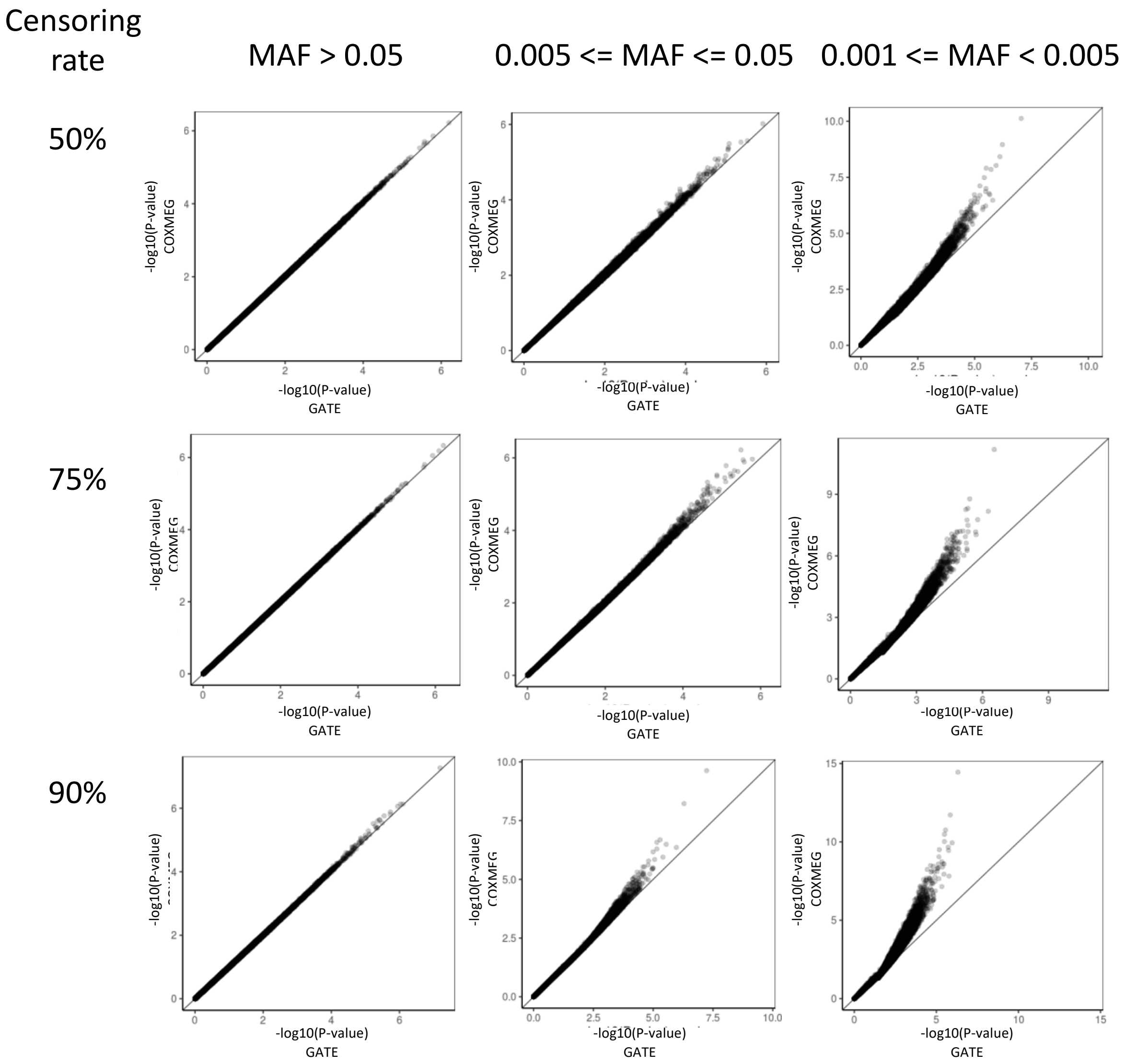

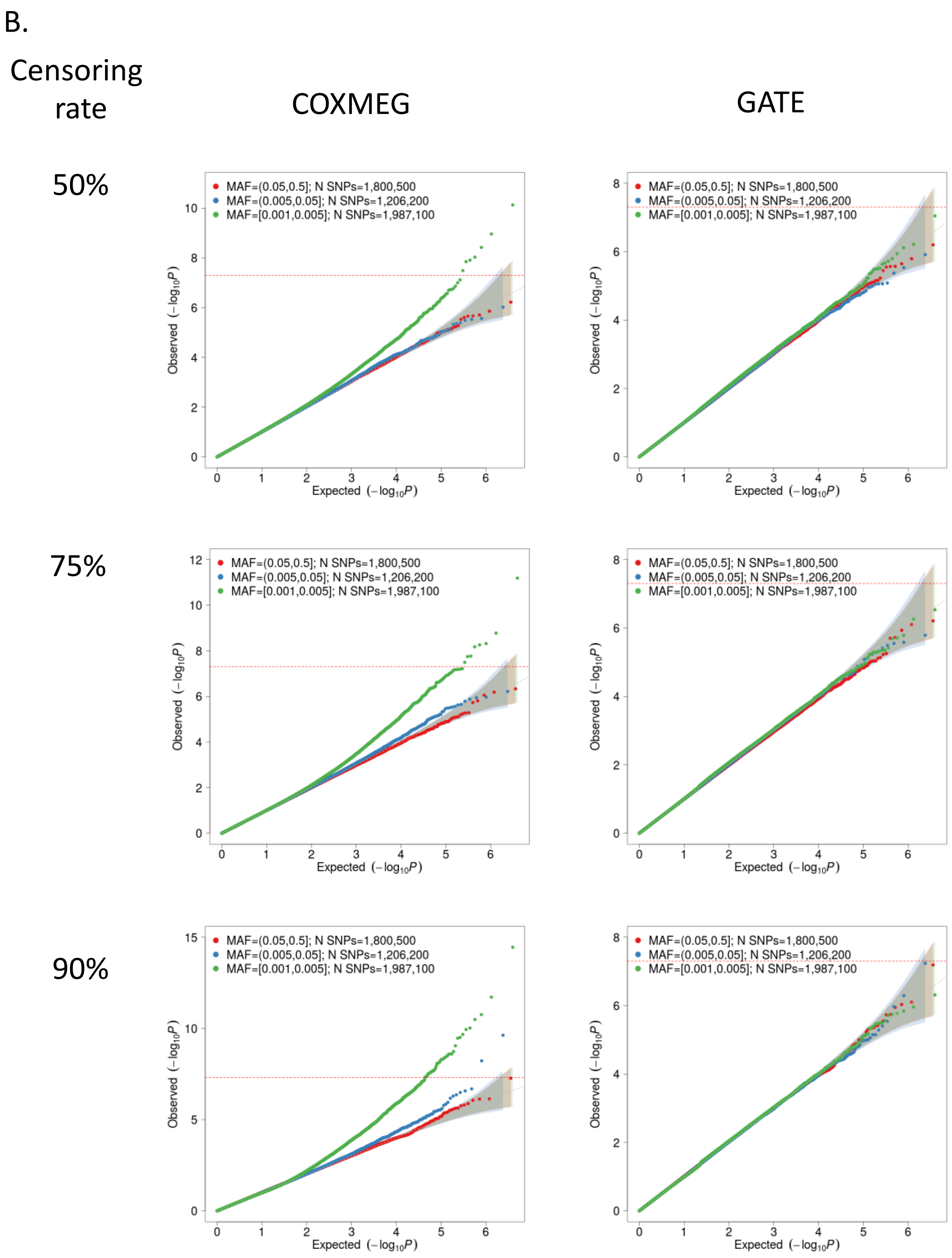
B.
Censoring rate
COXMEG
GATE
50%
75%
90%
MAF=(0.05,0.5]; N SNPs=1,800,500
MAF=(0.005,0.05]; N SNPs=1,206,200
MAF=[0.001,0.005]; N SNPs=1,987,100
Observed (−log10P)
Expected (−log10P)

C.

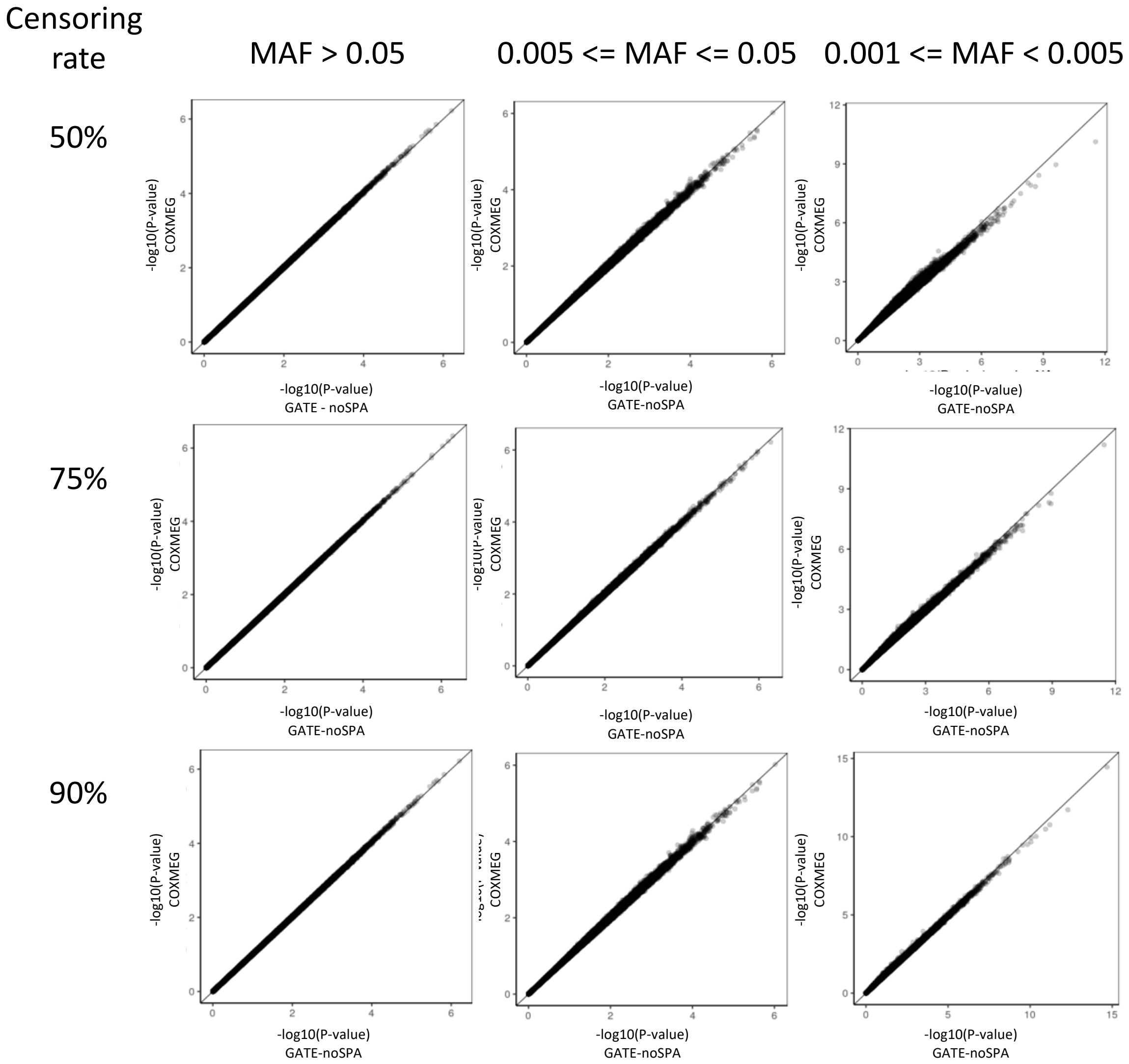

Censoring rate
MAF > 0.05
0.005 <= MAF <= 0.05
0.001 <= MAF < 0.005
50%
75%
90%
-log10(P-value) COXMEG
-log10(P-value) GATE - noSPA
-log10(P-value) GATE-noSPA

Supplementary Figure 2: Histogram of censoring rates of 871 PheCodes in the UK Biobank subjects with White British ancestry. The PheCodes are constructed based on the ICD9 and ICD10 codes and the associated diagnostic dates. Detailed description of the PheCode construction is available in the ONLINE METHODS section.

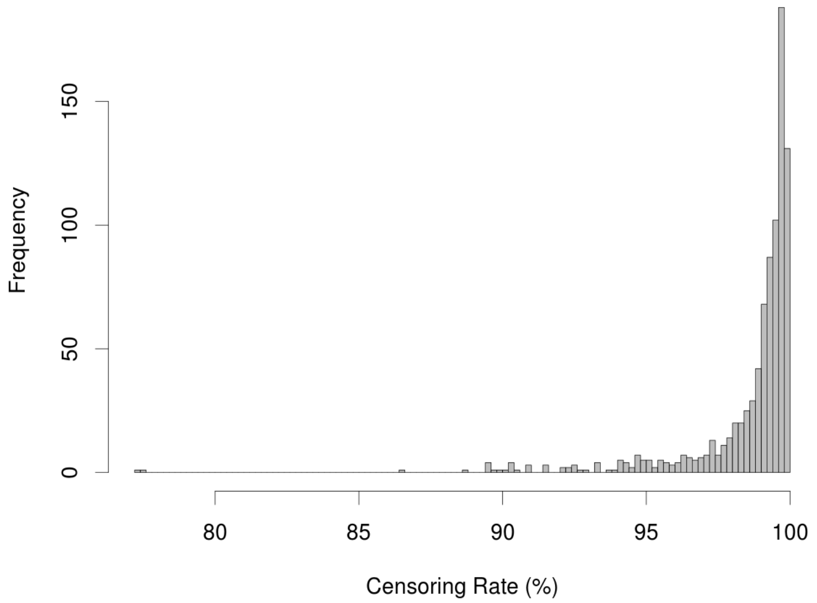

Supplementary Figure 3: Disease-free probability over-age by genotypes for loci LPA and CELSR2 for ischemic heart disease, FGFR2 and CASC16 for female breast cancer, MYOC and TMCO1 for glaucoma, and APOE e4 variant for AD. The red, green and blue lines represent the disease-free probability for alternate allele counts zero, one and two, respectively.

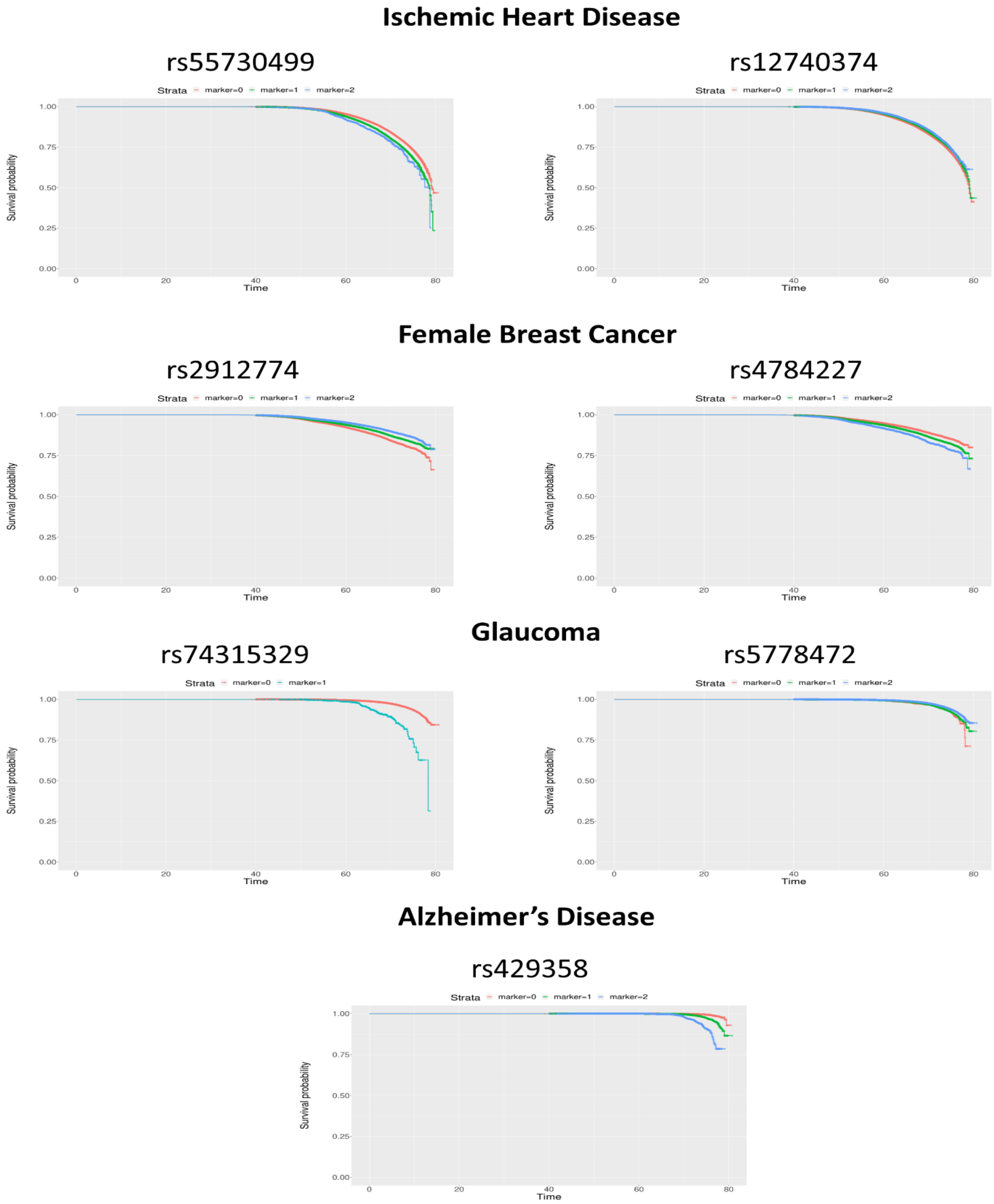

Supplementary Figure 4: Genome-wide association study of lifespan in the FinnGen Study (N events=15,152, N censored=203,244): A. Overall survival curve for lifespan B. GATE QQ plot (left) and Manhattan plot (right), C. SAIGE QQ plot (left) and Manhattan plot (right). 95% error bands around the nominal x=y diagonal line are also shown for each MAF category in panels B and C.

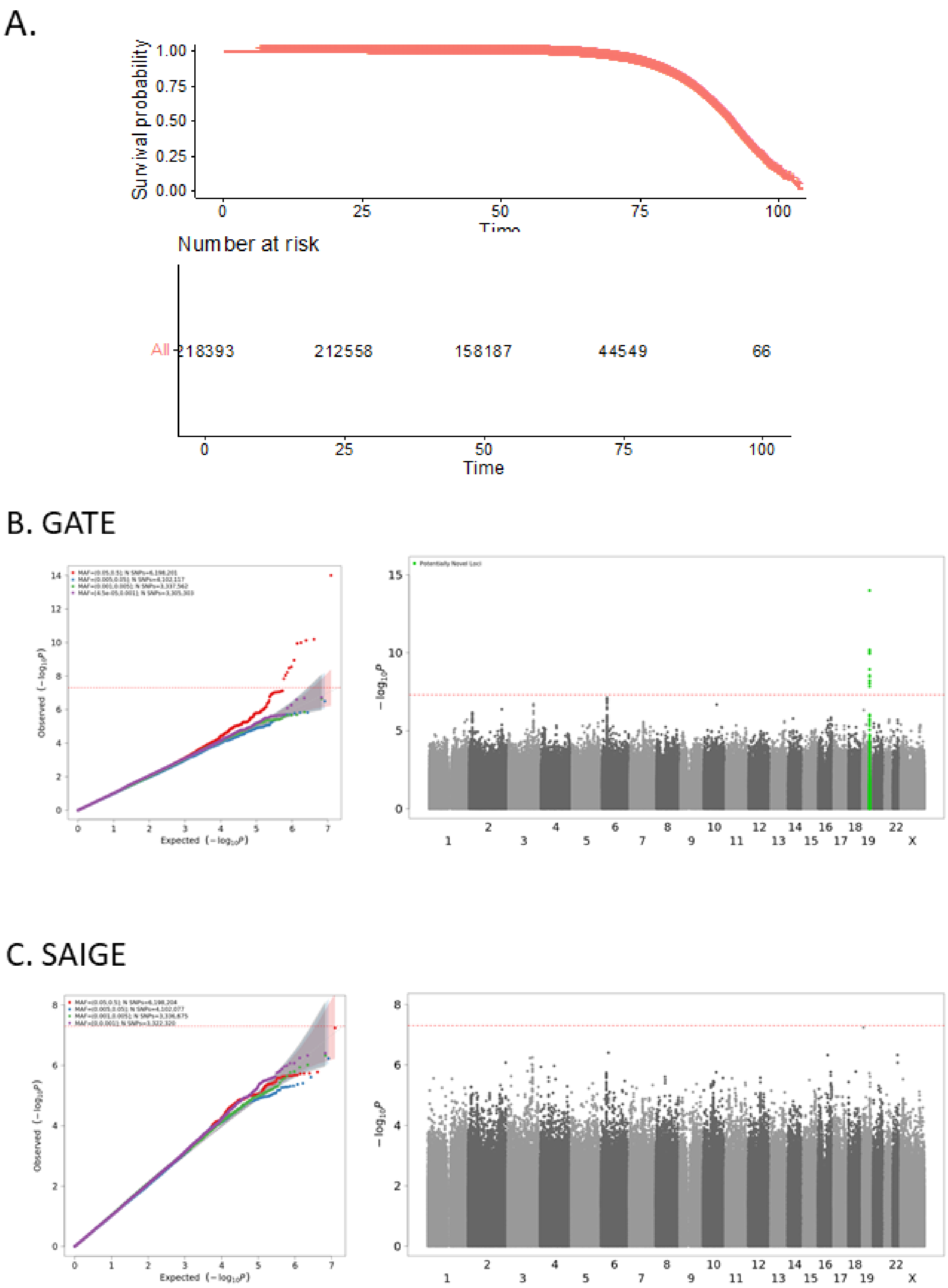

Supplementary Figure 5: Genome-wide association study of lifespan in the UK Biobank (N events=16,875, N censored=389,721), and meta-analysis results between the UK Biobank (N events=16,875, N censored=389,721) and the FinnGen Study (N events=15,152, N censored=203,244): A. GATE QQ plot (left) and Manhattan plot (right) in the UK Biobank, B. Meta-analysis Manhattan plot. 95% error bands around the nominal x=y diagonal line are also shown for each MAF category in panel A.

A. GATE on the UK Biobank

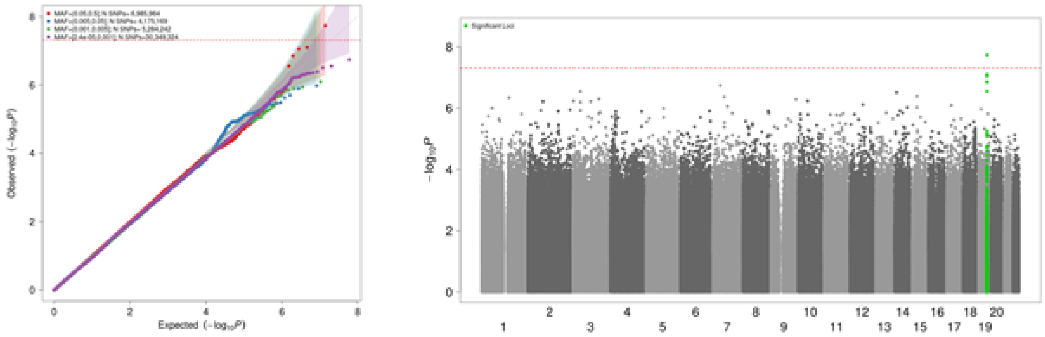

B. Meta-Analysis of the UK Biobank and Finngen Study

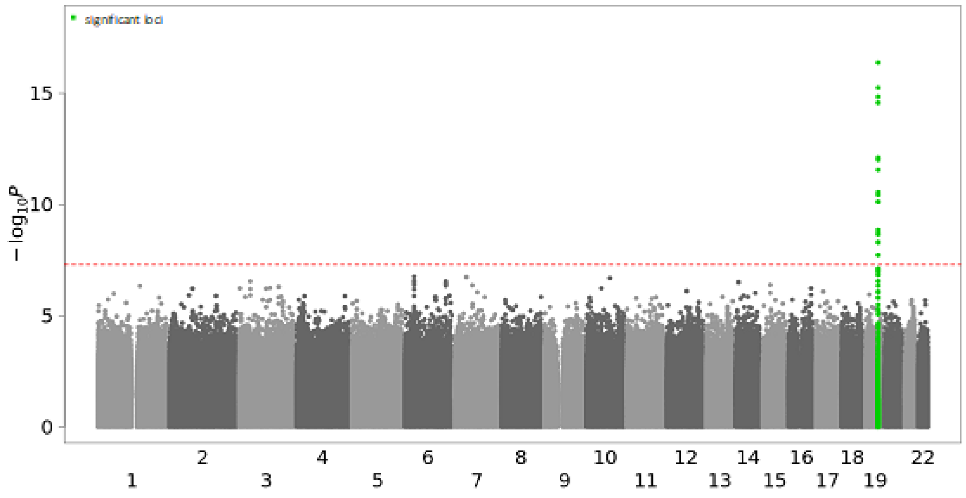

Supplementary Figure 6: Pedigree of families, each with 10 members, in the simulation study.

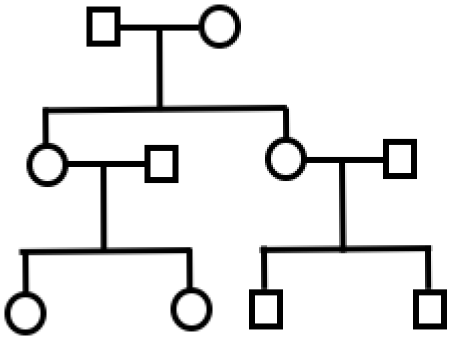

Supplementary Figure 7: Empirical type I error rates and 95% confidence intervals around the empirical type I error rates (horizontal bars) for GATE and GATE with no SPA estimated in simulation studies were plotted for censoring rates 50%. 75%, and 90%. A. based 9.4x10^{8} association tests. For each censoring rate, 100 data sets were simulated. Each data set contains 5,000 independent individuals and 500 families. Each family was simulated following the pedigree structure shown in Supplementary Figure 6. The variance component parameter τ = 0.1 and 0.25. Numbers were presented in Supplementary Table 3. B. based on 8.3x10^{8} association tests. or each censoring rate, 100 data sets were simulated. Each data set contains 10,000 randomly selected individuals with white British ancestry. The variance component parameter τ = 0.25. Numbers were presented in Supplementary Table 4.

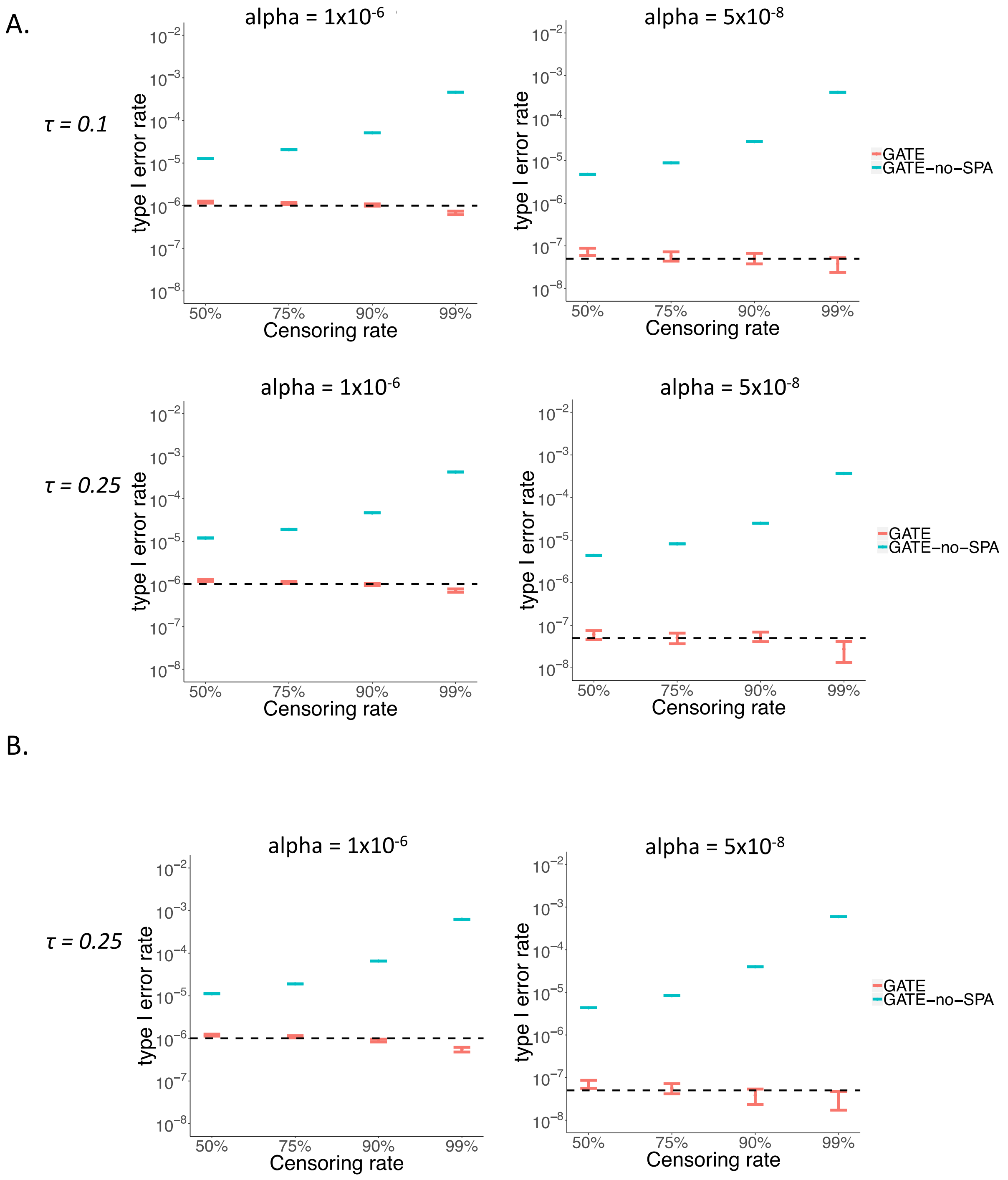

Supplementary Figure 8: Quantile-quantile plots stratified by minor allele frequencies (MAF) for randomly selected 10 million association tests from the simulation study for evaluating type I error rates

using 10,000 randomly selected individuals with white British ancestry (Supplementary Table 4). 95% error bands around the nominal x=y diagonal line are also shown for each MAF category.

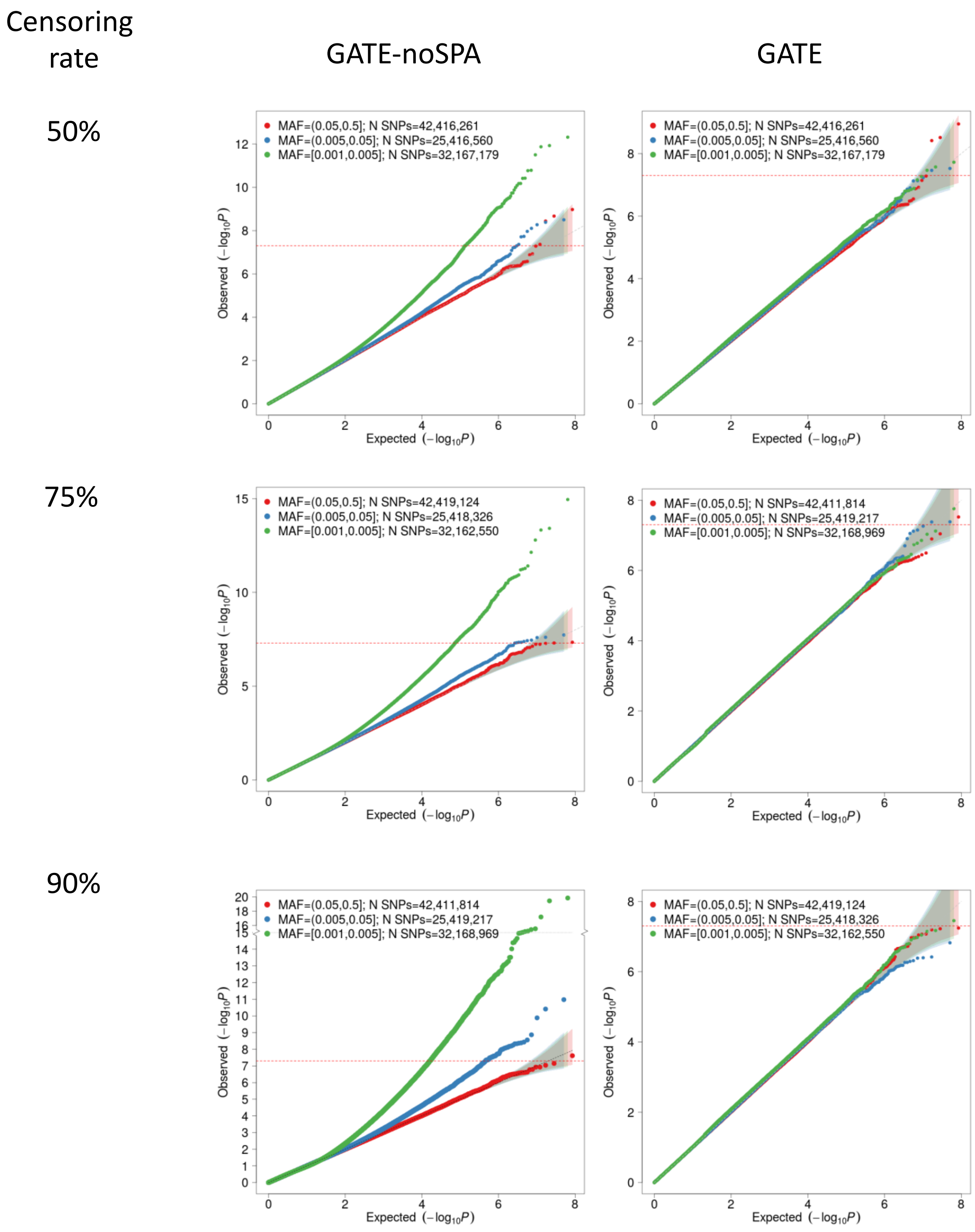

Supplementary Figure 9: Empirical power of GATE and COXMEG at the significance level α = 5×10^{-8}, when the variance component parameter τ = 0.25 and the censoring rate is 50%.

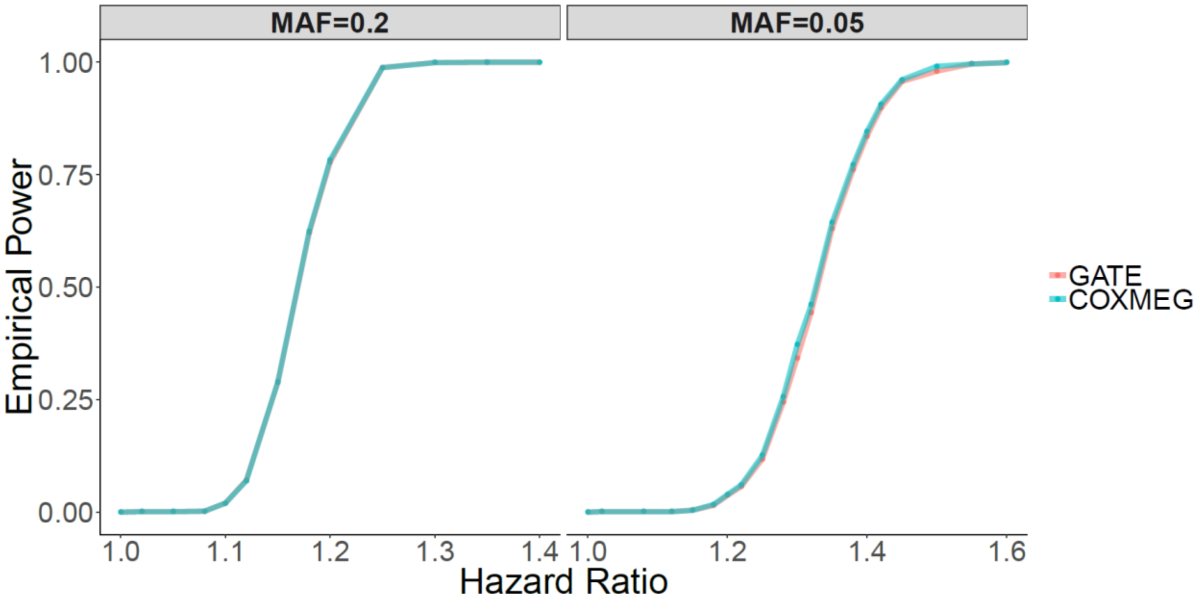

Supplementary Figure 10: Empirical power of GATE and SAIGE at the significance level α = 5×10^{-8} for TTE phenotypes with censoring rates 50% (A), 75% (B), and 95% (C).

A. Time-to-event outcome with censoring rate 50%

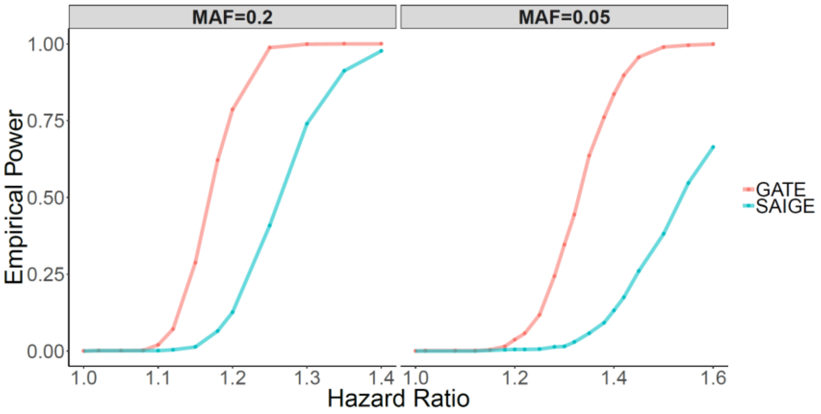

B. Time-to-event outcome with censoring rate 75%

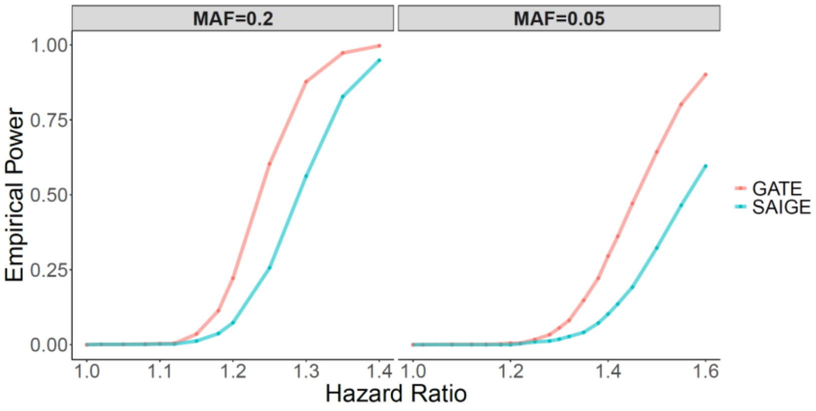

C. Time-to-event phenotype with censoring rate 95%

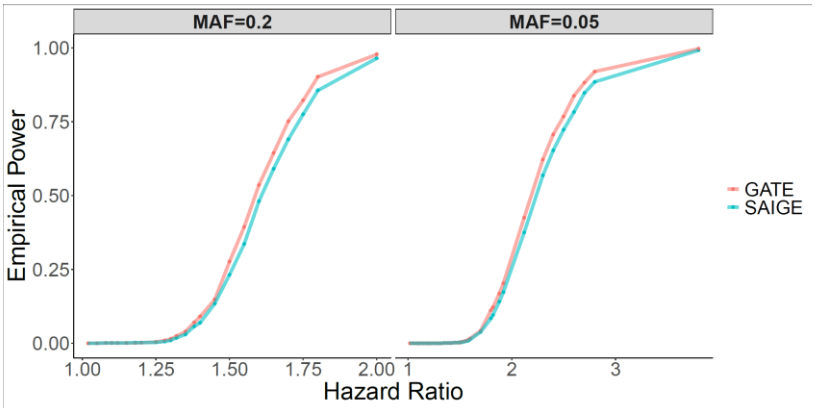

Supplementary Figure 11: Comparison of association p-values between using GATE and SAIGE for analysing the four time-to-event phenotypes based on the UK Biobank data. The x-axis represents the -log10 transformed p-values using SAIGE, and the y-axis represents the -log10 transformed p-values using GATE. Four phenotypes (ischemic heart disease, female breast cancer, glaucoma, and Alzheimer's disease) were analysed for association across 46 million imputed genetic variants with INFO ≥ 0.3 and MAC ≥20.

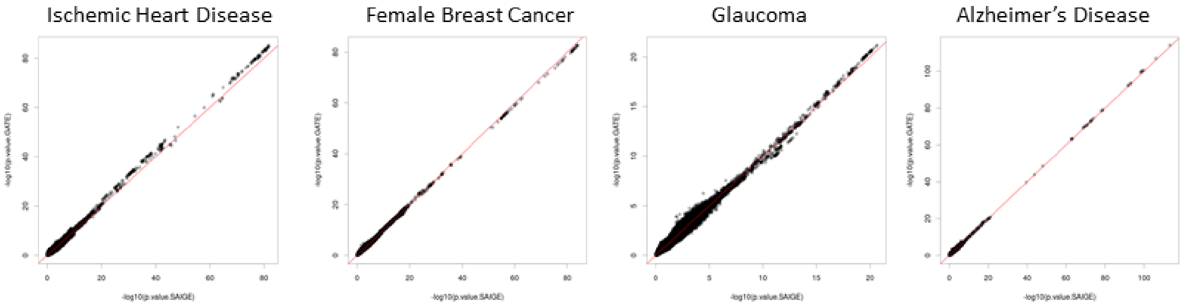

Supplementary Figure 12: Comparison of GATE association p-values between using different time-units for defining the event times of the four time-to-event phenotypes based on the UK Biobank data. Results were compared among the event and censoring times specified in nearest 1 month, 3 months, 6 months and 12 months time-units. Four phenotypes (ischemic heart disease, female breast cancer, glaucoma, and Alzheimer's disease) were analysed for association across 46 million imputed genetic variants with INFO ≥ 0.3 and MAC ≥ 20.

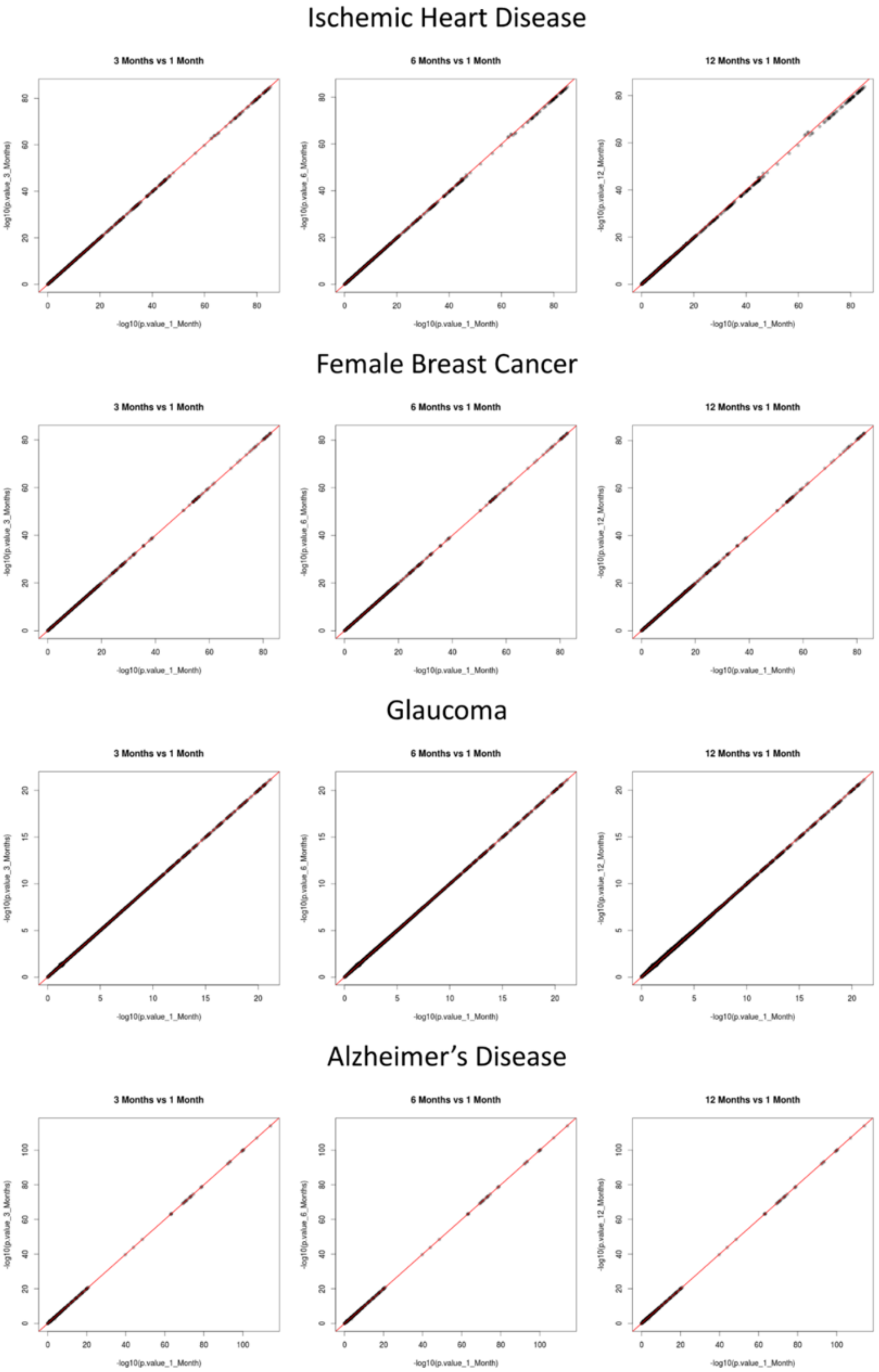

Supplementary Figure 13: Comparison of association p-values between using different number of markers for constructing the GRM in step 1 of GATE for analysing the four time-to-event phenotypes based on the

UK Biobank data. Results were compared between 93511 high-quality genotyped markers used by the UK Biobank research group for estimating kinship, and 245745 pruned markers with MAF>1%. Four phenotypes (ischemic heart disease, female breast cancer, glaucoma, and Alzheimer's disease) were analysed for association across 46 million imputed genetic variants with INFO ≥ 0.3 and MAC ≥20.

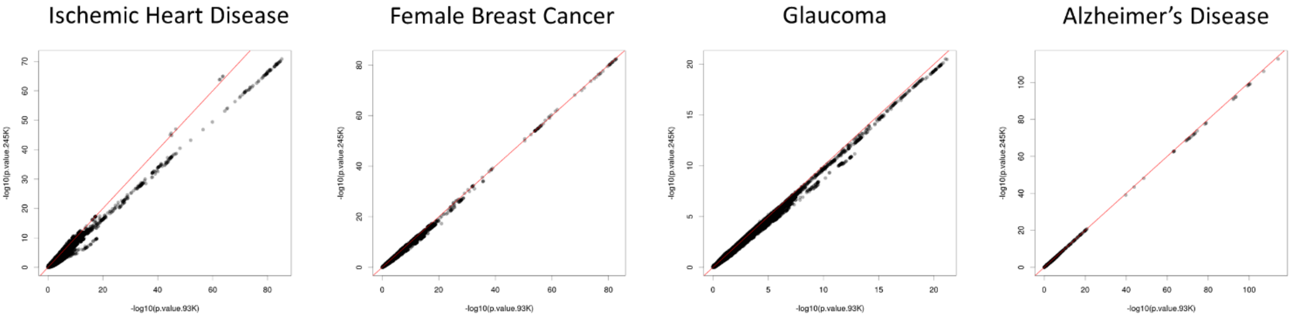

Supplementary Figure 14: Manhattan plots for GWAS of four binary phenotypes based the UK Biobank subjects with White British ancestry using GATE with A) 93,511 markers used in the GRM, and B) 245,745 markers used in the GRM. Four phenotypes (ischemic heart disease, female breast cancer, glaucoma, and Alzheimer's disease) were analysed for association across 46 million imputed genetic variants with INFO ≥ 0.3 and MAC ≥ 20.

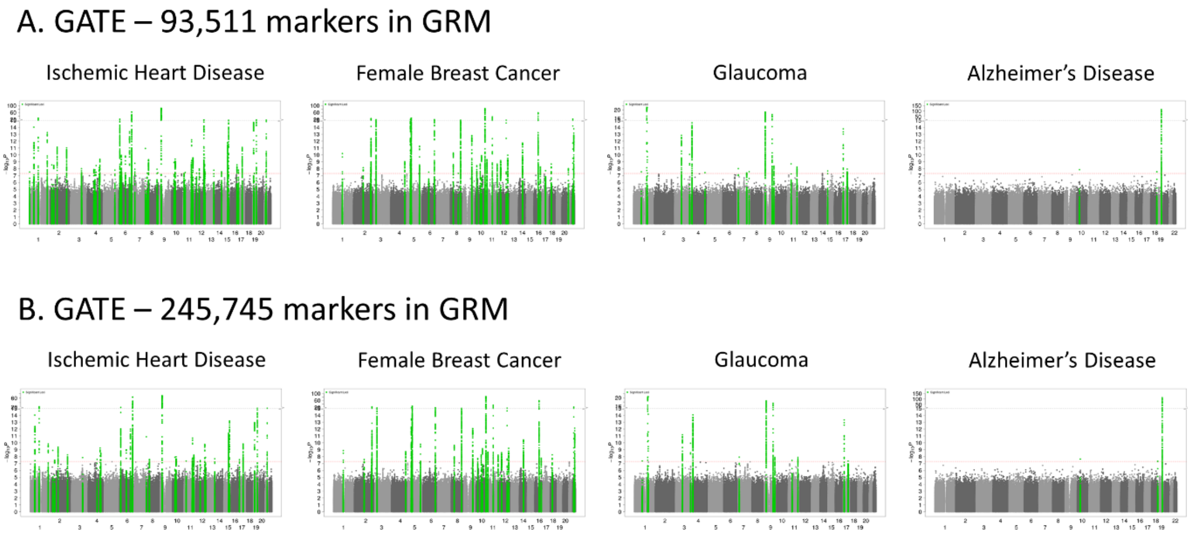

Supplementary Figure 15: Estimated variance ratios using different number of randomly selected markers for four phenotypes based on the UK Biobank subjects with White British ancestry using GATE.

For each choice of number of markers, we randomly selected the markers 50 times. The dotted lines represent the variance ratios with 500 randomly selected markers.

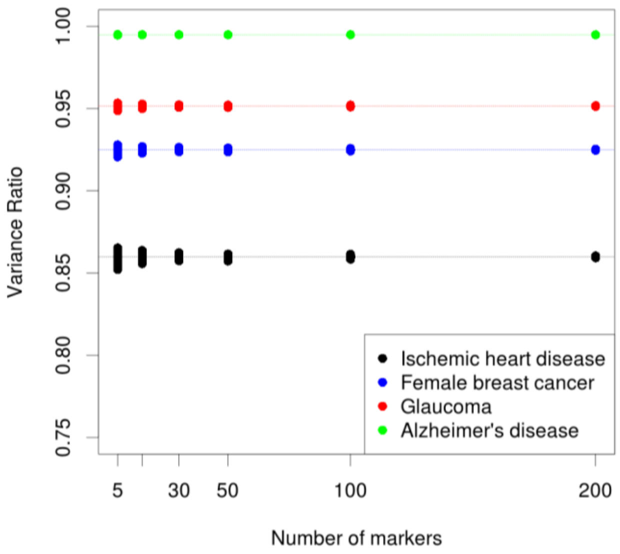

Efficient and accurate frailty model approach for genome-wide survival association analysis in large-scale biobanks - Supplementary Note

1 Derivation of the GATE Method

1.1 Derivation of the likelihood

Consider a set of N subjects where E_i and C_i denote the failure and censoring times respectively, for subject $i = 1, \ldots, N$. Let $T_i = \min(E_i, C_i)$, and $\Delta_i = I(E_i \leq C_i)$, where $I(.)$ denotes the indicator function. For each subject, we have the observed event status-time pair $(T_i = t_i, \Delta_i = \delta_i)$. We call t_i a failure time for subject i if $\delta_i = 1$, and a censoring time if $\delta_i = 0$. Without loss of generality, we assume t_is are in increasing order, i.e, $t_1 \leq t_2 \leq \ldots \leq t_N$.

Let X_i denote the $q \times 1$ vector of covariates (excluding the intercept) to adjust for, and G_i denote the genotype $(0, 1$, or $2)$ of the variant we are testing. Throughout this paper, quantities without a subscript is used to denote the vectors of corresponding quantities with subscripts (eg. $G = (G_1, \ldots, G_N)$), when it is clear from the context. Denote X to be the $n \times q$ covariate matrix with i-th row as $X_i^\top$. Then, in a frailty model, we denote the conditional hazard function for subject i at time t given the random effects b_is,

$$\lambda_i(t|b) = \lambda_0(t) \exp(\eta_i); \qquad \eta_i = \mathbf{X}_i^\top \beta + G_i \gamma + b_i,$$

and the survival function $S_i(t|b) = \exp(-\Lambda_0(t) \exp(\eta_i))$, where $\Lambda_0(t) = \int_0^t \lambda_0(u) du$ is the cumulative baseline hazard (CBH) function. Under multivariate Gaussian frailty, the random effects $\mathbf{b} \sim N(0, \tau \mathbf{V})$, where τ is the variance component, and $\mathbf{V}$ is the $N \times N$ genetic relationship matrix (GRM). We further assume that conditional on $\mathbf{b}$, the censoring is independent and non-informative of $\mathbf{b}$. Then the likelihood of the observed data is,

$$\begin{aligned} L(\lambda_0, \beta, \gamma, \tau) &= \int \prod_{i=1}^{N} \left[\lambda_i(t_i|b)^{\delta_i} S_i(t_i|b)\right] \frac{1}{|\tau V|^{1/2}} \exp\left(-\frac{1}{2}\mathbf{b}^\top (\tau V)^{-1} \mathbf{b}\right) d\mathbf{b} \\ &= \frac{1}{|\tau V|^{1/2}} \prod_{i=1}^{N} \lambda_0(t_i)^{\delta_i} \int \exp\left[\sum_{i=1}^{N} (\delta_i \eta_i - \Lambda_0(t_i)\exp(\eta_i)) - \frac{1}{2}\mathbf{b}^\top (\tau V)^{-1}\mathbf{b}\right] d\mathbf{b}. \end{aligned}$$

Let $f(\mathbf{b}) = \sum_{i=1}^{N} (\delta_i \eta_i - \Lambda_0(t_i)\exp(\eta_i)) - \frac{1}{2}\mathbf{b}^\top (\tau V)^{-1}\mathbf{b}$. Then we can approximate the integral using the Laplace approximation,

$$\int \exp f(\mathbf{b}) db \approx (2\pi)^{N/2} \left|-f''(\tilde{\mathbf{b}})\right|^{-1/2} \exp(f(\tilde{\mathbf{b}})),$$

where $\tilde{\mathbf{b}} = \arg_b \max f(\mathbf{b})$ is the solution to $f'(\mathbf{b}) = 0$. Therefore, the log-likelihood can be approximated by,

$$
\begin{aligned}
\ell((\lambda_0, \beta, \gamma, \tau) \approx & -\frac{1}{2}\log|\tau\mathbf{V}| + \sum_{i=1}^{N} \delta_i \log \lambda_0(t_i) - \frac{1}{2}\log\left|-f''(\tilde{\mathbf{b}})\right| + f(\tilde{\mathbf{b}}) \\
= & -\frac{1}{2}\log|\tau\mathbf{V}| - \frac{1}{2}\log\left|\sum_{i=1}^{N} \Lambda_0(t_i)\exp(\tilde{\eta}_i) + (\tau\mathbf{V})^{-1}\right| \\
& + \sum_{i=1}^{N}\left[\delta_i\left(\log\lambda_0(t_i) + \tilde{\eta}_i\right) - \Lambda_0(t_i)\exp(\tilde{\eta}_i)\right] - \frac{1}{2}\tilde{\mathbf{b}}^\top(\tau\mathbf{V})^{-1}\tilde{\mathbf{b}} \\
= & -\frac{1}{2}\log|\tau\mathbf{V}| - \frac{1}{2}\log\left|\tilde{\mathbf{W}} + (\tau\mathbf{V})^{-1}\right| \\
& + \sum_{i=1}^{N}\left[\delta_i\left(\log\lambda_0(t_i) + \tilde{\eta}_i\right) - \tilde{\mu}_i\right] - \frac{1}{2}\tilde{\mathbf{b}}^\top(\tau\mathbf{V})^{-1}\tilde{\mathbf{b}},
\end{aligned} \tag{1}
$$

where $\tilde{\eta}_i = \mathbf{X}_i^\top\beta + G_i\gamma + \tilde{b}_i$, $\tilde{\mu}_i = \Lambda_0(t_i)\exp(\tilde{\eta}_i)$, and $\tilde{W} = diag(\tilde{\mu}_i)$. The log-likelihood (1) with respect to β and τ is similar to the Poisson GLMM log-likelihood.[1,2] Following Breslow and Clayton,[2] assuming the weight matrix W changes slowly as a function of the mean, we can maximize the following penalized log-likelihood[3] given τ,

$$
\ell_p(\lambda_0, \beta, \gamma, b) = \sum_{i=1}^{N}\left[\delta_i\left(\log\lambda_0(t_i) + \eta_i\right) - \mu_i\right] - \frac{1}{2}\mathbf{b}^\top(\tau\mathbf{V})^{-1}\mathbf{b}, \tag{2}
$$

to obtain the maximum likelihood estimators (MLEs) $(\hat{\lambda}_0, \hat{\beta}, \hat{\gamma}, \hat{b}) = (\hat{\lambda}_0^\tau, \hat{\beta}^\tau, \hat{\gamma}^\tau, \hat{\mathbf{b}}^\tau)$, where $\hat{\mathbf{b}}^\tau = \tilde{\mathbf{b}}(\hat{\beta}^\tau)$, and $\mu_i = \Lambda_0(t_i)\exp(\eta_i)$.

1.2 Estimation of the baseline hazard, fixed effects and random effects given the variance component

Lets denote the unique failure times to be $t_1^* < t_2^* < \ldots < t_K^*$ in increasing order. Following Breslow's suggestion,[4] we assume the baseline hazard function to remain constant between successive event times, and according to Kalbfleisch and Prentice's[5] convention, we consider the censored observations to be censored at the previous failure time. In section 6 we show that the maximum likelihood estimation under these assumptions is equivalent to maximizing a partial likelihood.[6] Formally, lets denote $\lambda_0(t) = \alpha_i$, for $t_i^* \le t < t_{i+1}^*$. Then the cumulative hazard function can be written as a step function,

$$
\Lambda_0(t) = \sum_{j=1}^{i} \alpha_j\left(t_j^* - t_{j-1}^*\right), \quad t_i^* \le t < t_{i+1}^*,
$$

with the convention $t_0^* = t_0 = 0$. Then, using algebraic manipulations with the order of the summations, we can write,

$$
\sum_{i=1}^{N}\mu_i = \sum_{i=1}^{K}\left[\alpha_i(t_i^* - t_{i-1}^*)\sum_{j\in R(t_i^*)}\exp(\eta_j)\right],
$$

where $R(t_i) = \{j : t_j \geq t_i\}$ denotes the set of subjects at risk at time t_i. This definition of the at-risk set corresponds to Breslow's approximation when there are tied failure times.[7] We can express ℓ_p as a function of $(\alpha, \beta, \gamma, b)$, and obtain the score functions by taking derivatives of ℓ_p with respect to β, γ, b, and α_i-s,

$$\begin{aligned}
\frac{\partial \ell_p}{\partial \beta} &= \sum_{i=1}^{N} (\delta_i - \mu_i) X_i \\
\frac{\partial \ell_p}{\partial \gamma} &= \sum_{i=1}^{N} (\delta_i - \mu_i) G_i \\
\frac{\partial \ell_p}{\partial b} &= \sum_{i=1}^{N} (\delta_i - \mu_i) Z_i - (\tau \mathbf{V})^{-1}\mathbf{b} \\
\frac{\partial \ell_p}{\partial \alpha_i} &= \frac{d_i}{\alpha_i} - (t_i^* - t_{i-1}^*) \sum_{j \in R(t_i^*)} \exp(\eta_j),
\end{aligned} \tag{3}$$

where Z_i is an $N \times 1$ vector with i-th element 1 and rest 0s, and $d_i = \sum_{j:t_j = t_i^*} \delta_j$ is the number of failures at time t_i^*. Setting $\partial \ell_p / \partial \alpha_i = 0$, we obtain the MLEs,

$$\hat{\alpha}_i = d_i \left[(t_i^* - t_{i-1}^*) \sum_{j \in R(t_i^*)} \exp(\eta_j) \right]^{-1}.$$

This leads to the famous Breslow's estimator for the CBH,

$$\hat{\Lambda}_0(t) = \sum_{i=1}^{N} \frac{\delta_i I(t_i \leq t)}{\sum_{j \in R(t_i)} \exp(\eta_j)}.$$

For the score test of $H_0 : \gamma = 0$ vs $H_1 : \gamma \neq 0$, we only estimate the MLEs $(\hat{\alpha}, \hat{\beta}, \hat{\mathbf{b}})$ under H_0, setting $\gamma = 0$. Using similar techniques to estimate fixed effects parameters in Poisson GLMM, we denote the working outcome vector $\mathbf{Y} = \eta + W^{-1}(\delta - \mu)$, where $W = diag(\mu_i)$, $\eta = (\eta_1, \ldots, \eta_N)$, $\delta = (\delta_1, \ldots, \delta_N)$, and $\mu = (\mu_1, \ldots, \mu_N)$. Then, $\delta - \mu = W(Y - \eta) = W(Y - X\beta - Zb)$, and the score equations can be written as,

$$\begin{bmatrix} \mathbf{X}^\top \mathbf{W} \mathbf{X} & \mathbf{X}^\top \mathbf{W} \\ \mathbf{W} \mathbf{X} & \mathbf{W} + (\tau \mathbf{V})^{-1} \end{bmatrix} \begin{bmatrix} \beta \\ b \end{bmatrix} = \begin{bmatrix} \mathbf{X}^\top \mathbf{W} \mathbf{Y} \\ \mathbf{W} \mathbf{Y} \end{bmatrix}$$

Let $\mathbf{\Sigma} = \mathbf{W^{-1}} + \tau \mathbf{V}$, then

$$\begin{aligned}
\hat{\beta} &= \left(\mathbf{X}^\top \mathbf{\Sigma^{-1}} \mathbf{X} \right)^{\mathbf{-1}} \mathbf{X}^\top \mathbf{\Sigma^{-1}} \mathbf{Y} \\
\hat{\mathbf{b}} &= \tau \mathbf{V} \mathbf{\Sigma^{-1}} \left(\mathbf{Y} - \mathbf{X} \hat{\beta} \right).
\end{aligned}$$

1.2.1 Estimation of the variance component

Given $\hat{\alpha} = \hat{\alpha}(\tau), \hat{\beta} = \hat{\beta}(\tau)$ estimated, from (1) the log-likelihood of the variance component can be derived as,

$$\ell(\hat{\alpha}(\tau), \hat{\beta}(\tau), \gamma = 0, \tau) = -\frac{1}{2} log|\mathbf{\Sigma}| - \frac{\mathbf{1}}{\mathbf{2}} \mathbf{Y}^\top \mathbf{P} \mathbf{Y},$$

where $\mathbf{P} = \mathbf{\Sigma^{-1}} - \mathbf{\Sigma}^\top \mathbf{X} \left(\mathbf{X}^\top \mathbf{\Sigma^{-1}} \mathbf{X}\right)^{\mathbf{-1}} \mathbf{X}^\top \mathbf{\Sigma^{-1}}$. We maximize the corresponding restricted maximum-likelihood (REML),

$$\ell_R(\hat{\alpha}(\tau), \hat{\beta}(\tau), \gamma = 0, \tau) = -\frac{1}{2} log|\mathbf{\Sigma}| - \frac{1}{2} log|\mathbf{X}^\top \mathbf{\Sigma^{-1}} \mathbf{X}| - \frac{\mathbf{1}}{\mathbf{2}} \mathbf{Y}^\top \mathbf{P} \mathbf{Y}.$$

The score function with respect to τ are given by,

$$\mathbf{U}_\tau = \frac{\partial \ell_R(\hat{\alpha}(\tau), \hat{\beta}(\tau), \gamma = 0, \tau)}{\partial \tau} = \frac{1}{2} \left[\mathbf{Y}^\top \mathbf{PVPY} - tr(\mathbf{PV})\right].$$

The corresponding observed information function, and the expected information function are given by

$$\mathbf{J}_\tau = -\frac{\partial^2 \ell_R(\hat{\alpha}(\tau), \hat{\beta}(\tau), \gamma = 0, \tau)}{\partial \tau^2} = -\frac{1}{2} tr(\mathbf{PVPV}) + \mathbf{Y}^\top \mathbf{PVPVPY},$$

$$E\left(\mathbf{J}_\tau\right) = E\left[-\frac{\partial^2 \ell_R(\hat{\alpha}(\tau), \hat{\beta}(\tau), \gamma = 0, \tau)}{\partial \tau^2}\right] = \frac{1}{2} tr(\mathbf{PVPV}),$$

respectively. Evaluating both observed and expected information functions involves computationally expensive trace computations. To avoid the trace computations, the average information is used in the AI-REML[1,8,9] algorithm. The average information is expressed as the average of J_τ and $E(J_\tau)$,

$$\mathbf{AI}_\tau = \frac{1}{2} \mathbf{Y}^\top \mathbf{PVPVPY}.$$

1.2.2 Algorithm to fit the null mixed model

The null model fitting algorithm can be summarized as,

1. Fit a Poisson linear model wth $\tau = 0$ to get initial estimates $\hat{\beta}^{(0)}$ and working outcome vector $Y^{(0)}$.

2. Calculate Breslow's estimator $\hat{\Lambda}_0^{(0)}(t)$.

3. At the i-th step, update $\hat{\tau}$ using $\hat{\tau}^{(i)} = \hat{\tau}^{(i-1)} + \left\{\mathbf{AI}_\tau\Big|_{\tau=\hat{\tau}^{(i-1)}}\right\}^{-1} \left\{\mathbf{U}_\tau\Big|_{\tau=\hat{\tau}^{(i-1)}}\right\}$.

4. Update $\hat{\beta}, \hat{b}, \hat{\alpha}$ using $\mathbf{Y}$ and $\hat{\tau}^{(i)}$.

5. Update $\mathbf{Y}$ and $\hat{\Lambda}_0(t)$ using $\hat{\beta}^{(i)}, \hat{b}^{(i)}, \hat{\alpha}^{(i)}, \hat{\tau}^{(i)}$.

6. Repeat steps 2–5, until $\max\left\{\frac{\left|\hat{\beta}^{(i)} - \hat{\beta}^{(i-1)}\right|}{\left|\hat{\beta}^{(i)}\right| + \left|\hat{\beta}^{(i-1)}\right|}, \frac{\left|\hat{\tau}^{(i)} - \hat{\tau}^{(i-1)}\right|}{\left|\hat{\tau}^{(i)}\right| + \left|\hat{\tau}^{(i-1)}\right|}\right\} \leq tolerance$.

1.3 Score test

The score test statistic under the null hypothesis is given by,

$$T = \frac{\partial l}{\partial \gamma}\Big|_{\left(\hat{\beta},\hat{b},\gamma=0,\hat{\alpha},\hat{\tau}\right)} = \mathbf{G}^\top(\delta - \hat{\mu}) = \tilde{\mathbf{G}}^\top(\delta - \hat{\mu}),$$

where $\tilde{\mathbf{G}} = \mathbf{G} - \tilde{\mathbf{X}}\left(\tilde{\mathbf{X}}^\top\hat{\mathbf{W}}\tilde{\mathbf{X}}\right)^{\mathbf{-1}}\tilde{\mathbf{X}}^\top\hat{\mathbf{W}}\mathbf{G}$ is the covariate-and-intercept adjusted genotype vector, and $\tilde{\mathbf{X}} = \begin{bmatrix}1 & X\end{bmatrix}$ is the intercept-augmented covariate matrix. The information matrix corresponding to the score equations in (3) is given by,

$$\mathbf{I}(\beta,\gamma,b,\alpha) = \begin{bmatrix} \mathbf{X}^\top\mathbf{W}\mathbf{X} & \mathbf{X}^\top\mathbf{W}\mathbf{G} & \mathbf{X}^\top\mathbf{W} & \mathbf{B}^{(\mathbf{X})\top} \\ \mathbf{G}^\top\mathbf{W}\mathbf{X} & \mathbf{G}^\top\mathbf{W}\mathbf{G} & \mathbf{G}^\top\mathbf{W} & \mathbf{B}^{(\mathbf{G})\top} \\ \mathbf{W}\mathbf{X} & Z^\top\mathbf{W}\mathbf{G} & \mathbf{W} + (\tau\mathbf{V})^{\mathbf{-1}} & \mathbf{B}^{(\mathbf{Z})\top} \\ \mathbf{B}^{(\mathbf{X})} & \mathbf{B}^{(\mathbf{G})} & \mathbf{B}^{(\mathbf{Z})} & \mathbf{B}^{(\lambda)} \end{bmatrix},$$

where,

$$\begin{aligned}
\mathbf{B}^{(\lambda)} &= diag\left(-\frac{\partial^2 \ell_p}{\partial \alpha_i}\right) = diag\left(d_i/\alpha_i^2\right) \\
\mathbf{B}_{\bullet\mathbf{i}}^{(\mathbf{X})\top} &= -\frac{\partial^2 \ell_p}{\partial \alpha_i \partial \beta^\top} = (t_i^* - t_{i-1}^*)\sum_{j\in R(t_i^*)} \exp(\eta_j)X_j \\
\mathbf{B}_{\bullet\mathbf{i}}^{(\mathbf{G})\top} &= -\frac{\partial^2 \ell_p}{\partial \alpha_i \partial \gamma^\top} = (t_i^* - t_{i-1}^*)\sum_{j\in R(t_i^*)} \exp(\eta_j)G_j \\
\mathbf{B}_{\bullet\mathbf{i}}^{(\mathbf{Z})\top} &= -\frac{\partial^2 \ell_p}{\partial \alpha_i \partial b^\top} = (t_i^* - t_{i-1}^*)\sum_{j\in R(t_i^*)} \exp(\eta_j)Z_j.
\end{aligned}$$

A_{ij} and $A_{\bullet i}$ denote the (i,j)-th element and i-th column of a matrix A, respectively. Then, the variance of the score statistic under H_0 is given by,

$$Var_{H_0}(T) = \left(\mathbf{I}(\hat{\beta},\hat{\mathbf{b}},\gamma=\mathbf{0},\hat{\alpha})^{\mathbf{-1}}\right)_{\mathbf{22}} = \mathbf{G}^\top\hat{\mathbf{Q}}\mathbf{G} = \tilde{\mathbf{G}}^\top\hat{\mathbf{Q}}\tilde{\mathbf{G}},$$

where $\hat{\mathbf{Q}} = \hat{\mathbf{S}}^{\mathbf{-1}} - \hat{\mathbf{S}}^{\mathbf{-1}}\mathbf{X}\left(\mathbf{X}^\top\mathbf{S}^{\mathbf{-1}}\mathbf{X}\right)^{\mathbf{-1}}\mathbf{X}^\top\hat{\mathbf{S}}^{\mathbf{-1}}$, $\hat{\mathbf{S}} = (\hat{\mathbf{W}} - \hat{\mathbf{U}})^{\mathbf{-1}} + \hat{\tau}\mathbf{V}$. The matrix $\hat{\mathbf{U}}$ is defined as,

$$\hat{\mathbf{U}} = \sum_{i=1}^{K} \frac{d_i}{(v_i^\top 1)^2} v_i v_i^\top = \mathbf{\Gamma}\mathbf{R}^\top\mathbf{D}\mathbf{R}\mathbf{\Gamma},$$

$$\mathbf{D} = diag\left\{\frac{d_i}{\left(\sum_{j\in R(t_i^*)}\exp(\hat{\eta}_j)\right)^2}\right\}_{i=1}^{K}, \qquad \mathbf{\Gamma} = diag\left\{\exp(\hat{\eta}_i)\right\}_{i=1}^{N},$$

$$R_{ij} = \begin{cases} 0, & \text{if } j \notin R(t_i^*) \\ 1, & \text{if } j \in R(t_i^*) \end{cases}, \quad \text{for } i = 1,\ldots,K, j = 1,\ldots,N,$$

where $\mathbf{v}_i$ is an n-dimensional vector with elements $v_{ij} = 0$ if $j \notin R(t_i^*)$, and $v_{ij} = \exp(\hat{\eta}_j)$ if $j \in R(t_i^*)$.

2 Approaches to reduce computation and memory cost

To obtain $Var_{H_0}(T) = \tilde{\mathbf{G}}^\top \hat{\mathbf{Q}} \tilde{\mathbf{G}}$, we need to compute quantities of the form $\hat{\mathbf{S}}^{-\mathbf{1}}\mathbf{a}$, where $\mathbf{a}$ is a vector. The standard computation technique of inverting $\hat{\mathbf{S}}$ (computation cost $O(N^3)$) and multiplying $\hat{\mathbf{S}}^{-\mathbf{1}}$ with $\mathbf{a}$ can be extremely time consuming when N is large. On top of that, it will require the storage of $\hat{\mathbf{S}}^{-\mathbf{1}}$ (memory cost $O(N^2)$) which can have extremely high memory requirement. In order to reduce the computation and memory cost, we implemented several strategies similar to BOLT-LMM[10] and SAIGE.[9] Firstly, instead of storing the $N \times N$ GRM which can cost $4N(N+1)$ bytes if stored using double precision floating point numbers, we only store the raw genotypes as binary vectors which only costs $NM/4$ bytes, where M is the number of markers used to calculate the GRM. We calculate the elements of the GRM from the raw genotype vectors only when they are needed. Secondly, to compute quantities of the form $\hat{\mathbf{S}}^{-\mathbf{1}}\mathbf{a}$, we implemented the pre-conditioned conjugate gradient[11] (PCG) method, which computes $\hat{\mathbf{S}}^{-\mathbf{1}}\mathbf{a} = \mathbf{b}$ by solving the linear system of equations $\hat{\mathbf{S}}\mathbf{b} = \mathbf{a}$. Thirdly, since $\mathbf{U} = \mathbf{\Gamma R}^\top \mathbf{DR\Gamma}$ has a low rank decomposition with $rank(\mathbf{U}) = K$, the number of unique failure times, to calculate $(\mathbf{W} - \mathbf{U})^{-\mathbf{1}}\mathbf{G}$ in the PCG steps, we leverage the Woodbury identity decomposition

$$(\mathbf{W} - \mathbf{U})^{-\mathbf{1}}\mathbf{G} = \mathbf{W}^{-\mathbf{1}}\mathbf{G} - \mathbf{W}^{-\mathbf{1}}\mathbf{\Gamma R^T}(-\mathbf{D}^{-\mathbf{1}} + \mathbf{R\Gamma W}^{-\mathbf{1}}\mathbf{\Gamma R^T})^{-\mathbf{1}}\mathbf{R\Gamma W}^{-\mathbf{1}}\mathbf{G}.$$

The matrix $(-\mathbf{D}^{-\mathbf{1}} + \mathbf{R\Gamma W}^{-\mathbf{1}}\mathbf{\Gamma R^T})$ is a $K \times K$ matrix which can be inverted easily when K is small, or when K is also larger, PCG can be used to obtain $(-\mathbf{D}^{-\mathbf{1}} + \mathbf{R\Gamma W}^{-\mathbf{1}}\mathbf{\Gamma R^T})^{-\mathbf{1}}\mathbf{R\Gamma W}^{-\mathbf{1}}\mathbf{G}$.

Similar to SAIGE, we further implemented the Hutchinson's randomized trace estimation[12,13] for calculating $tr(\mathbf{PV})$. We also implemented multithreaded parallel computation for the matrix-vector multiplications in the PCG steps using Intel Threading Building Block (TBB) from the RcppParallel[14] package.

3 Variance ratio approximation

Computation of the variance of the score statistic $Var_{H_0}(T) = \tilde{\mathbf{G}}^\top \hat{\mathbf{Q}} \tilde{\mathbf{G}}$ requires calculating $\hat{\mathbf{Q}}\tilde{\mathbf{G}}$ repeatedly for all markers, which is computationally expensive. To avoid calculating $\hat{\mathbf{Q}}\tilde{\mathbf{G}}$ for all the markers, we estimate the variance ratio $\hat{r} = \tilde{\mathbf{G}}^\top \hat{\mathbf{Q}} \tilde{\mathbf{G}} / \tilde{\mathbf{G}}^\top \hat{\mathbf{W}} \tilde{\mathbf{G}}$ using a small set of markers, and then approximate the variance of the score statistic for all markers by $\hat{r}\tilde{\mathbf{G}}^\top \hat{\mathbf{W}} \tilde{\mathbf{G}}$ in step 2. This saves substantial computation time since $\hat{\mathbf{W}}$ is a diagonal matrix. Such variance ratio approximation approaches were previously used in various linear[10,15,16] and logistic mixed effects models[9] to speed up computation. Here, we provide a theoretical justification for why such an approximation works.

Let $\mathbf{P}_{\tilde{\mathbf{X}}} = \tilde{\mathbf{X}}(\tilde{\mathbf{X}}^\top \hat{\mathbf{W}} \tilde{\mathbf{X}})^{-\mathbf{1}} \tilde{\mathbf{X}}^\top \hat{\mathbf{W}}$ be the weighted projection matrix for the intercept-augmented covariate matrix $\tilde{\mathbf{X}}$. Let $E(G_i) = \mu_g$ and the covariance matrix of $\mathbf{G}$ is given by $\sigma_g^2 \mathbf{\Psi}$, where $\mathbf{\Psi}$ is the correlation matrix of $\mathbf{G}$. When the elements of $\mathbf{G}$ follows the $Bin(2, p_g)$ distribution, then $\mu_g = 2p_g$, and $\sigma_g^2 = \sqrt{2p_g(1-p_g)}$. The matrix $\mathbf{\Psi}$ represents the kinship matrix, however exact characterization of $\mathbf{\Psi}$ is not needed for this proof. Then, $E(\tilde{\mathbf{G}}) = \mu_g(\mathbf{I} - \mathbf{P}_{\tilde{\mathbf{X}}})\mathbf{1} = 0$, and $Cov(\mathbf{G}) = \sigma_g^2(\mathbf{I} - \mathbf{P}_{\tilde{\mathbf{X}}})\mathbf{\Psi}(\mathbf{I} - \mathbf{P}_{\tilde{\mathbf{X}}})^\top$, where $\mathbf{1}$ is the $N \times N$ vector of all element equal to unity. We scale both the numerator and denominator of the variance ratio by N^{-1} so that

they don't blow to infinity when looked at individually. Then, for the numerator,

$$E(N^{-1}\tilde{G}^\top \hat{Q}\tilde{G}) = \frac{\sigma_g^2}{N} tr\left[\hat{Q}(I - P_{\tilde{X}})\Psi(I - P_{\tilde{X}})^\top\right] = \frac{\sigma_g^2}{N} tr(\hat{Q}\Psi),$$

since $(I - P_{\tilde{X}})^\top \hat{Q}(I - P_{\tilde{X}}) = \hat{Q}$. Similarly, for the denominator,

$$E(N^{-1}\tilde{\mathbf{G}}^\top \hat{\mathbf{W}}\tilde{\mathbf{G}}) = \frac{\sigma_g^2}{N} tr\left[\hat{\mathbf{W}}(\mathbf{I} - \mathbf{P}_{\tilde{\mathbf{X}}})\boldsymbol{\Psi}(\mathbf{I} - \mathbf{P}_{\tilde{\mathbf{X}}})^\top\right] = \frac{\sigma_g^2}{N} tr\left[\hat{\mathbf{W}}(\mathbf{I} - \mathbf{P}_{\tilde{\mathbf{X}}})\boldsymbol{\Psi}\right],$$

since $(\mathbf{I} - \mathbf{P}_{\tilde{\mathbf{X}}})^\top \hat{\mathbf{W}}(\mathbf{I} - \mathbf{P}_{\tilde{\mathbf{X}}}) = \hat{\mathbf{W}}(\mathbf{I} - \mathbf{P}_{\tilde{\mathbf{X}}})$. Therefore, as the eigenvalues of $\hat{\mathbf{Q}}, \hat{\mathbf{W}}, \boldsymbol{\Psi}$ are bounded, and the distribution of $\mathbf{G}$ has bounded support, the variances of the numerator and the denominator terms are both $O(N^{-1})$, and the variance ratio converges to,

$$\hat{r} = \frac{\tilde{\mathbf{G}}^\top \hat{\mathbf{Q}}\tilde{\mathbf{G}}}{\tilde{\mathbf{G}}^\top \hat{\mathbf{W}}\tilde{\mathbf{G}}} \xrightarrow{p} \frac{lim_{N\to\infty}\left\{N^{-1}tr(\hat{\mathbf{Q}}\boldsymbol{\Psi})\right\}}{lim_{N\to\infty}\left\{N^{-1}tr\left[\hat{\mathbf{W}}(\mathbf{I} - \mathbf{P}_{\tilde{\mathbf{X}}})\boldsymbol{\Psi}\right]\right\}}.$$

The ratio on the right-hand side is constant across all markers as the individual limits in the numerator and denominator exist and are bounded away from zero.

Our software first estimates the variance ratio using 30 randomly selected markers, and then adds more markers with increments of ten until the coefficient of variation (CV) is smaller than 0.001. We performed a sensitivity analysis on the number of markers used to calculate the variance ratio using the UK Biobank data on white British subjects. For the analysis of the four exemplary phenotypes described in this paper, we used different number of markers ($N = 5, 15, 30, 50, 100, 200$) to estimate the variance ratios. For each choice of number of markers, we selected the markers randomly 50 times and the variance ratio estimates are presented in Supplementary Figure 15. The results show that the variance ratio estimates remain overall very stable when ≥ 30 markers are used, and the variation decreases with increasing number of markers.

4 Using saddlepoint approximation[17] (SPA) for the null distribution of the score statistic

In traditional score tests, the distribution of the score statistic under H_0 is approximated by a Normal distribution, which uses the first two moments, mean and variance. This approach can perform poorly in the tail regions, especially if the underlying distribution is highly skewed when the studying event is very rare or the testing genetic variant has a very low minor allele count (MAC). Here, similar to what has been applied in the logistic mixed models[9,18–20] previously, we use the SPA to approximate the null distribution of the score statistic to obtain accurate p-values. Based on the likelihood (1), we derive the SPA of $T_{adj} = \tilde{\mathbf{G}}^\top(\delta - \hat{\mu})/\sqrt{\hat{r}\tilde{\mathbf{G}}^\top\hat{\mathbf{W}}\tilde{\mathbf{G}}}$ as a weighted sum of independent Poisson random variables given b. The approximated cumulant generating function (CGF) of T_{adj} and it's derivatives

are given by,

$$K(\xi;\hat{\mu}) = \sum_{i=1}^{N} \hat{\mu}_i (e^{\tilde{G}_i c \xi} - \tilde{G}_i c \xi - 1),$$
$$K'(\xi;\hat{\mu}) = \sum_{i=1}^{N} \hat{\mu}_i \tilde{G}_i c (e^{\tilde{G}_i c \xi} - 1),$$
$$K''(\xi;\hat{\mu}) = \sum_{i=1}^{N} \hat{\mu}_i \tilde{G}_i^2 c^2 e^{\tilde{G}_i c \xi},$$

where $c = (\tilde{G}^\top W \tilde{G})^{-1/2}$. To calculate the probability that $T_{adj} < s$, where s is the observed test statistic, we use the following formula

$$Pr(T < s) = \Phi\left\{w + \frac{1}{w} log\left(\frac{v}{w}\right)\right\},$$

where $w = sign(\hat{\xi})[2\{\hat{\xi}s - K(\hat{\xi})\}]^{\frac{1}{2}}$, $v = \hat{\xi}[K''(\hat{\xi})]^{\frac{1}{2}}$, $\hat{\xi}$ is the solution of $K'(\hat{\xi}) = s$, and Φ is the standard normal distribution function.

5 Effect size estimation

Since our method only fits the model under the null hypothesis of no association, it cannot provide the effect size estimates as part of the model fitting process. Instead, to rapidly estimate the effect sizes, we follow a similar approach used in EMMAX,[21] GRAMMAR-Gamma,[15] and SAIGE[9] using the parameter estimates from the null model. Our genetic effect size estimate is given by $\hat{\gamma} = \left(\tilde{\mathbf{G}}^\top \hat{\mathbf{Q}} \tilde{\mathbf{G}}\right)^{-\mathbf{1}} \tilde{\mathbf{G}}^\top \hat{\mathbf{Q}}(\delta - \hat{\mu})$. Notice that this estimate can also be expressed as $\hat{\gamma} = T/Var_{H_0}(T)$ where T is the score test statistic, and derived assuming the standardized Wald test statistic to be equal to the standardized score test statistic. In section 3, we showed that $Var_{H_0}(T) = \tilde{\mathbf{G}}^\top \hat{\mathbf{Q}} \tilde{\mathbf{G}} \approx \hat{r} \tilde{\mathbf{G}}^\top \hat{\mathbf{W}} \tilde{\mathbf{G}}$. Therefore, $\hat{\gamma} = T/\hat{r}\tilde{\mathbf{G}}^\top \hat{\mathbf{W}} \tilde{\mathbf{G}}$, which can be estimated using the already estimated quantities T,$\hat{r}$, and $\tilde{\mathbf{G}}^\top \hat{\mathbf{W}} \tilde{\mathbf{G}}$. The standard errors can be estimated by inverting the p-values. The standard error of $\hat{\gamma}$, $SE(\hat{\gamma}) = |\hat{\gamma}/z|$, where z is the Z-score corresponding to the two-sided association p-value.

6 Equivalence of penalized full and partial likelihood-based estimates

For unrelated samples, the equivalence between maximizing the partial likelihood, and maximizing the full likelihood with Breslow's estimator plugged in as the estimate of the CBH function has been shown by Breslow[4] in the proportional hazard model. Here, we show that given the variance component τ, maximizing ℓ_p (see (2)) assuming that the baseline hazard to be constant between any two consecutive failure times, i.e., estimating the CBH function

using Breslow's estimator, is equivalent to maximizing the penalized partial log-likelihood as described in Ripati et al.,[6]

$$\ell_{pp}\left(\beta,\gamma,b\right)=\sum_{i=1}^{N}\delta_i\left(\eta_i-\log\sum_{j\in R(t_i)}\exp(\eta_j)\right)-\frac{1}{2}\mathbf{b}^\top(\tau\mathbf{V})^{-\mathbf{1}}\mathbf{b}. \tag{4}$$

Plugging in $\hat{\Lambda}_0(t)$ into the score equations corresponding to β,γ in (3), and using algebraic manipulations with the ordering of the summations, we get,

$$\frac{\partial\ell_p}{\partial\beta}=\sum_{i=1}^{n}\delta_i\left(X_i-\frac{\sum_{j\in R(t_i)}\exp(\eta_j)X_j}{\sum_{j\in R(t_i)}\exp(\eta_j)}\right),$$
$$\frac{\partial\ell_p}{\partial\gamma}=\sum_{i=1}^{n}\delta_i\left(G_i-\frac{\sum_{j\in R(t_i)}\exp(\eta_j)G_j}{\sum_{j\in R(t_i)}\exp(\eta_j)}\right).$$

We observe that the expressions of the score functions are the same as the score functions from the partial likelihood $\partial\ell_{pp}/\partial\beta$,$\partial\ell_{pp}/\partial\gamma$. Equivalence of the information matrices can also be shown similarly.

7 Sensitivity analysis on the number of markers used to construct the GRM

We performed a sensitivity analysis on the number of markers used to construct the GRM in step 1 of GATE. We compared association results between using 93511 high-quality genotyped markers that were used by the UK Biobank research group to calculate kinship,[22] and 245745 pruned (500kb window, sliding step-size 50 markers, $r^2 < 0.2$) genotyped markers with MAF ≥ 0.01. We compared the association results between these two marker-sets for the analysis of four example phenotypes in the UK Biobank data on 46 million imputed variants. Manhattan plots (Supplementary Figure 14) were similar between the two marker-sets, and the scatter plots of the p-values (Supplementary Figure 13) show highly correlated association p-values. P-values using 245745 markers were generally slightly smaller than the p-values using 93511 markers, especially for ischemic heart disease. Similar observation was also made for the analysis of the binary disease status for this phenotype.[9]

8 Benchmarking GATE and COXMEG

To compare the computational performances of GATE, COXMEG-Score, and COXMEG-Sparse, we applied these methods on randomly sampled subsets of different sample sizes from 408,582 UK Biobank subjects with White British ancestry. Top four principal components, birth year and sex were adjusted for in the analysis, and 200,000 randomly selected variants out of 46 million genetic variants (imputation info ≥ 0.3 and MAC ≥ 20) were tested for association with overall lifespan (16,375 events, 389,721 censored). COXMEG version 1.0.11 and GATE version 0.40.1 were used for this comparison.

8.1 Commands for running GATE

To set up the time-unit and sample-size:

```
eventTimeBinSize=1        #for 1 year as time-unit
N=200000        #Number of subjects
i=1        #Replication ID. We used i=1,...,5
```

The arguments to run GATE step 1 were:

```
--eventTimeBinSize=$eventTimeBinSize
--phenoFile=pheno/pheno.365.$N.$i.txt
--outputPrefix=output/pheno.365.$N.$i
--nThreads=30
--plinkFile=geno/ukb_auto_v2_WB_inRelatedness
--phenoCol=casecontrol
--covarColList=PC1,PC2,PC3,PC4,birthYear,sex
--eventTimeCol=survtime
--sampleIDColinphenoFile=IID
--traitType=survival
```

The arguments to run GATE step 2 were:

```
--GMMATmodelFile=output/pheno.365.$N.$i.rda
--varianceRatioFile=output/pheno.365.$N.$i.varianceRatio.txt
--bgenFile=geno/ukb_imp_chr21_v3_365.$N.$i.bgen
--bgenFileIndex=geno/ukb_imp_chr21_v3_365.$N.$i.bgen.bgi
--sampleFile=geno/bgensamples_365.$N.$i.txt
```

Here, the `bgenFile` was created by randomly selecting 200,000 SNPs with imputation info ≥ 0.3 and MAC ≥ 20.

8.2 Commands for creating the dense and sparse GRM from the genotypes

```
#Read the phenotype file
    pheno<-read.table(paste0("pheno/pheno.365.",N,".",rep,".txt"),header=T)
#Read the genotypes using the BEDMatrix package
    mat<-BEDMatrix("geno/ukb_auto_v2_WB_inRelatedness")[paste0(pheno$IID,
        "_",pheno$IID),]
#Impute missing genotypes using mean imputation
    freq<-colMeans(mat,na.rm=T)/2
    ina <- which(is.na(mat))
    if ( length(ina) > 0 )  mat[ina] <- 2*freq[(ina-1)%/%nrow(mat)+1]
#Scaling and centering the genotype matrix
    mat<-(t(mat)-2*freq)/sqrt(2*freq*(1-freq))
#Calculating the dense GRM
```

```
    GRM<-t(mat)%*%mat/nrow(mat)
#Calculating the sparse GRM
    sparseGRM<-GRM
    sparseGRM[which(sparseGRM<0.05)]<-0
    sparseGRM<-as(sparseGRM,"dgCMatrix")
#Save the dense and sparse GRMs as RData files
    save(GRM,file=paste0("pheno_coxmeg/GRM.",N,".",rep,".RData"))
    save(sparseGRM,file=paste0("pheno_coxmeg/sparseGRM.",N,".",rep,".RData"))
```

The sparse GRM is stored in the sparse `dgCMatrix` format. The sizes of the dense and sparse GRMs in terms of hard disk storage space (megabytes - MB) and sparsity rates are as below. The median values of the corresponding metric out of five replications are reported.

Number of subjects	Dense storage (MB)	Sparse storage (MB)	Sparsity Rate
5000	182	0.117	$5042/2.5 \times 10^7 = 0.02\%$
10000	730	0.245	$10172/10^8 = 0.01\%$
20000	2922	0.494	$20694/4 \times 10^8 = 0.005\%$

8.3 Commands for running COXMEG-Score

```
#Load the dense GRM
    load(paste0("pheno_coxmeg/GRM.",N,".",rep,".RData"))
#Run the null model
    re=coxmeg_plink(
        pheno=paste0("pheno_coxmeg/pheno.365.",N,".",rep,".txt"),
        corr=GRM,
        spd=FALSE,
        type='dense',
        cov_file=paste0("pheno_coxmeg/cov.365.",N,".",rep,".txt"),
        verbose=TRUE
        )
#Run the association test
    coxmeg_plink(
        pheno=paste0("pheno_coxmeg/pheno.365.",N,".",rep,".txt"),
        bed=paste0("geno/ukb_imp_chr21_v3_365.",N,".",rep),
        tmp_dir="coxmeg_temp",
        corr=GRM,
        spd=FALSE,
        type='dense',
        cov_file=paste0("pheno_coxmeg/cov.365.",N,".",rep,".txt"),
        tau=re$tau,
        score=TRUE,
        verbose=TRUE
        )
```

8.4 Commands for running COXMEG-Sparse

```
#Load the dense GRM
    load(paste0("pheno_coxmeg/sparseGRM.",N,".",rep,".RData"))
#Run the null model
    re=coxmeg_plink(
        pheno=paste0("pheno_coxmeg/pheno.365.",N,".",rep,".txt"),
        corr=sparseGRM,
        spd=FALSE,
        type='sparse',
        cov_file=paste0("pheno_coxmeg/cov.365.",N,".",rep,".txt"),
        verbose=TRUE
        )
#Run the association test
    coxmeg_plink(
        pheno=paste0("pheno_coxmeg/pheno.365.",N,".",rep,".txt"),
        bed=paste0("geno/ukb_imp_chr21_v3_365.",N,".",rep),
        tmp_dir="coxmeg_temp",
        corr=sparseGRM,
        spd=FALSE,
        type='sparse',
        cov_file=paste0("pheno_coxmeg/cov.365.",N,".",rep,".txt"),
        tau=re$tau,
        score=FALSE,
        verbose=TRUE
        )
```

8.5 Computational resource requirements for steps 1 and 2

The computation time and memory requirements for the null model fitting (step 1) and association test (step 2) are as below. In addition to the sample size, the computation time for GATE in step 1 depends on other factors such as the number of steps required for the preconditioned conjugate gradient (PCG) method to converge and estimation of the variance component, especially when the sample-size is small. This explains the non-monotonic nature of the median computation time for GATE step 1 as the sample size increases in the low sample size regime, however, the mean computation times are still monotonically increasing with sample size.

Table 1: Projected computation time and memory usage for GATE, COXMEG-Score, and COXMEG-Sparse across different sample sizes. Benchmarking was performed for the genome-wide association study (GWAS) of lifespan based on randomly subsampled data from UK Biobank White British ancestry subjects. Association tests (step 2) were performed on 200,000 randomly selected markers with imputation INFO ≥ 0.3, with the filtering criteria of minor allele count (MAC) ≥ 20, and the computation times were projected for testing 46 million variants. The reported run times are medians (means inside parentheses) of five runs, each with randomly sampled subjects with different randomization seeds.

	Number of	Step 1		Step 2	
Method	subjects	Time (hours)	Memory (GB)	Time (hours)	Memory (GB)
GATE	5000	0.114 (0.189)	0.36	26.635 (29.177)	0.25
	10000	0.707 (0.512)	0.54	30.730 (30.080)	0.24
	20000	0.488 (0.634)	0.74	33.794 (35.374)	0.24
	50000	1.190 (1.444)	1.44	57.579 (55.465)	0.28
	100000	6.081 (4.639)	2.91	80.059 (79.927)	0.34
	200000	11.592 (12.570)	5.55	135.217 (138.504)	0.46
	408582	31.285 (31.907)	10.60	287.135 (298.015)	0.87
COXMEG-Score	5000	0.029 (0.029)	1.80	191.625 (187.138)	1.70
	10000	0.118 (0.118)	7.99	737.053 (745.665)	6.02
	20000	0.555 (0.555)	32.75	3355.525 (3322.719)	23.58
COXMEG-Sparse	5000	0.002 (0.002)	0.44	356.429 (358.061)	0.47
	10000	0.005 (0.005)	1.55	746.870 (747.809)	1.08
	20000	0.022 (0.021)	5.95	1412.454 (1414.219)	5.88

Supplementary References

[1] Han Chen, Chaolong Wang, Matthew p Conomos, Adrienne m Stilp, Zilin Li, Tamar Sofer, Adam a Szpiro, Wei Chen, John m Brehm, Juan c Celedón, Susan Redline, George j Papanicolaou, and Timothy a Thornton. Control for population structure and relatedness for binary traits in genetic association studies via logistic mixed models. *The American Journal of Human Genetics*, 98(4):653–666, 2016.

[2] N. E Breslow and D. G Clayton. Approximate inference in generalized linear mixed models. *Journal of the American Statistical Association*, 88(421):9–25, 1993.

[3] Peter J. Green. Penalized likelihood for general semi-parametric regression models. *International Statistical Review / Revue Internationale de Statistique*, 55(3):245–259, 1987.

[4] N.E. Breslow. Discussion of the paper by d. r. cox. *Journal of the Royal Statistical Society. Series B (Methodological)*, 34:216–217, 1972.

[5] J. D Kalbfleisch. *The statistical analysis of failure time data.* Wiley series in probability and mathematical statistics. Wiley, New York, 1980.

[6] Samuli Ripatti and Juni Palmgren. Estimation of multivariate frailty models using penalized partial likelihood. *Biometrics*, 56(4):1016–1022, 2000.

[7] Terry M. Therneau. *Modeling Survival Data: Extending the Cox Model.* Statistics for Biology and Health. Springer New York : Springer, New York, NY, 2000.

[8] Arthur R. Gilmour, Robin Thompson, and Brian R. Cullis. Average information reml: An efficient algorithm for variance parameter estimation in linear mixed models. *Biometrics*, 51(4):1440–1450, 1995.

[9] Wei Zhou, Jonas B Nielsen, Lars G Fritsche, Rounak Dey, Maiken E Gabrielsen, Brooke N Wolford, Jonathon Lefaive, Peter Vandehaar, Sarah A Gagliano, Aliya Gifford, Lisa A Bastarache, Wei-Qi Wei, Joshua C Denny, Maoxuan Lin, Kristian Hveem, Hyun Min Kang, Goncalo R Abecasis, Cristen J Willer, and Seunggeun Lee. Efficiently controlling for case-control imbalance and sample relatedness in large-scale genetic association studies. *Nature genetics*, 50(9):1335–1341, 2018.

[10] Po-Ru Loh, George Tucker, Brendan K Bulik-Sullivan, Bjarni J Vilhjálmsson, Hilary K Finucane, Rany M Salem, Daniel I Chasman, Paul M Ridker, Benjamin M Neale, Bonnie Berger, Nick Patterson, and Alkes L Price. Efficient bayesian mixed-model analysis increases association power in large cohorts. *Nature genetics*, 47(3):284, 2015.

[11] S Tsuruta, I Misztal, and I Strandén. Use of the preconditioned conjugate gradient algorithm as a generic solver for mixed-model equations in animal breeding applications. *Journal of animal science*, 79(5):1166–1172, 2001.

[12] M.F Hutchinson. A stochastic estimator of the trace of the influence matrix for laplacian smoothing splines. *Communications in Statistics - Simulation and Computation*, 19(2):433–450, 1990.

[13] Haim Avron and Sivan Toledo. Randomized algorithms for estimating the trace of an implicit symmetric positive semi-definite matrix. *Journal of the ACM (JACM)*, 58(2):1–34, 2011.

[14] JJ Allaire, Romain Francois, Kevin Ushey, Gregory Vandenbrouck, Marcus Geelnard, and Intel. *RcppParallel: Parallel Programming Tools for 'Rcpp'*, 2019. R package version 4.4.4.

[15] Gulnara R Svishcheva, Tatiana I Axenovich, Nadezhda M Belonogova, Cornelia M Van Duijn, and Yurii S Aulchenko. Rapid variance components–based method for whole-genome association analysis. *Nature Genetics*, 44(10):1166, 2012.

[16] Longda Jiang, Zhili Zheng, Ting Qi, Kathryn Kemper, Naomi Wray, Peter Visscher, and Jian Yang. A resource-efficient tool for mixed model association analysis of large-scale data. *Nature Genetics*, 51(12):1749–2, 2019.

[17] H. E. Daniels. Saddlepoint approximations in statistics. *The Annals of Mathematical Statistics*, 25(4):631–650, 1954.

[18] Rounak Dey, Ellen M Schmidt, Goncalo R Abecasis, and Seunggeun Lee. A fast and accurate algorithm to test for binary phenotypes and its application to phewas. *The American Journal of Human Genetics*, 101(1):37–49, 2017.

[19] Rounak Dey, Jonas B. Nielsen, Lars G. Fritsche, Wei Zhou, Huanhuan Zhu, Cristen J. Willer, and Seunggeun Lee. Robust meta-analysis of biobank-based genome-wide association studies with unbalanced binary phenotypes. *Genetic Epidemiology*, 43(5):462–476, 2019.

[20] Wenjian Bi, Zhangchen Zhao, Rounak Dey, Lars G Fritsche, Bhramar Mukherjee, and Seunggeun Lee. A fast and accurate method for genome-wide scale phenome-wide g x e analysis and its application to uk biobank. *The American Journal of Human Genetics*, 105(6):1182–1192, 2019.

[21] Hyun Min Kang, Jae Hoon Sul, Susan K Service, Noah A Zaitlen, Sit-Yee Kong, Nelson B Freimer, Chiara Sabatti, and Eleazar Eskin. Variance component model to account for sample structure in genome-wide association studies. *Nature genetics*, 42(4):348–354, 2010.

[22] Clare Bycroft, Colin Freeman, Desislava Petkova, Gavin Band, Lloyd T Elliott, Kevin Sharp, Allan Motyer, Damjan Vukcevic, Olivier Delaneau, Jared Oapos;Connell, Adrian Cortes, Samantha Welsh, Alan Young, Mark Effingham, Gil McVean, Stephen Leslie, Naomi Allen, Peter Donnelly, and Jonathan Marchini. The uk biobank resource with deep phenotyping and genomic data. *Nature (London)*, 562(7726):203–209, 2018.
