# Supplementary material for: Efficient and accurate frailty model approach for genome-wide survival association analysis in large-scale biobanks
Source: Nat Commun. 2022 Sep 16;13:5437. doi: 10.1038/s41467-022-32885-x (PMC9481565; doi:10.1038/s41467-022-32885-x)
Supplement: Supplementary file 3 — Description of Additional Supplementary Files [file 41467_2022_32885_MOESM3_ESM.pdf]

Description of Additional Supplementary Files

File Name: Supplementary Data 1
Description: Top genome-wide significant variants (α = 5E-08) in different loci based on GATE for four TTE phenotypes based on the UK Biobank data of subjects with White British ancestry and the Finngen study. For any variant with $p < 5E-08$, we extend upstream and downstream by 1Mb, then merge the overlapping regions together to define the locus and report the variant that has the smallest p-value in each locus. Asterisk (*) represents that the locus was genome-wide significant using GATE, but no locus within 1Mb neighbourhood of it was found to be significant using SAIGE. Genomic coordinates are based on NCBI Build 37/UCSC hg19.

File Name: Supplementary Data 2
Description: Summary information of 18 loci that were significant (α = 5E-08) based on GATE, but not significant based on SAIGE, for the four TTE phenotypes in the UK Biobank data of subjects with White British ancestry. Genomic coordinates are based on NCBI Build 37/UCSC hg19.
