# Supplementary material for: Efficient and accurate frailty model approach for genome-wide survival association analysis in large-scale biobanks
Source: Nat Commun. 2022 Sep 16;13:5437. doi: 10.1038/s41467-022-32885-x (PMC9481565; doi:10.1038/s41467-022-32885-x)
Supplement: Supplementary file 6 — Reporting Summary [file 41467_2022_32885_MOESM6_ESM.pdf]

nature portfolio

Corresponding author(s): Xihong Lin, Benjamin Neale, Mark Daly

Last updated by author(s): Jul 11, 2022

Reporting Summary

Nature Portfolio wishes to improve the reproducibility of the work that we publish. This form provides structure for consistency and transparency in reporting. For further information on Nature Portfolio policies, see our Editorial Policies and the Editorial Policy Checklist.

Statistics

For all statistical analyses, confirm that the following items are present in the figure legend, table legend, main text, or Methods section.

n/a	Confirmed	
☐	☒	The exact sample size (*n*) for each experimental group/condition, given as a discrete number and unit of measurement
☐	☒	A statement on whether measurements were taken from distinct samples or whether the same sample was measured repeatedly
☐	☒	The statistical test(s) used AND whether they are one- or two-sided<br>*Only common tests should be described solely by name; describe more complex techniques in the Methods section.*
☐	☒	A description of all covariates tested
☐	☒	A description of any assumptions or corrections, such as tests of normality and adjustment for multiple comparisons
☐	☒	A full description of the statistical parameters including central tendency (e.g. means) or other basic estimates (e.g. regression coefficient) AND variation (e.g. standard deviation) or associated estimates of uncertainty (e.g. confidence intervals)
☐	☒	For null hypothesis testing, the test statistic (e.g. *F*, *t*, *r*) with confidence intervals, effect sizes, degrees of freedom and *P* value noted<br>*Give P values as exact values whenever suitable.*
☒	☐	For Bayesian analysis, information on the choice of priors and Markov chain Monte Carlo settings
☐	☒	For hierarchical and complex designs, identification of the appropriate level for tests and full reporting of outcomes
☐	☒	Estimates of effect sizes (e.g. Cohen's *d*, Pearson's *r*), indicating how they were calculated

Our web collection on statistics for biologists contains articles on many of the points above.

Software and code

Policy information about availability of computer code

Data collection	No software was used.
Data analysis	GATE v0.42 (https://github.com/weizhou0/GATE), COXMEG v1.0.11 (https://cran.r-project.org/web/packages/coxmeg/index.html).

For manuscripts utilizing custom algorithms or software that are central to the research but not yet described in published literature, software must be made available to editors and reviewers. We strongly encourage code deposition in a community repository (e.g. GitHub). See the Nature Portfolio guidelines for submitting code & software for further information.

Data

Policy information about availability of data

All manuscripts must include a data availability statement. This statement should provide the following information, where applicable:

- Accession codes, unique identifiers, or web links for publicly available datasets
- A description of any restrictions on data availability
- For clinical datasets or third party data, please ensure that the statement adheres to our policy

The GWAS results using GATE for 871 time-to-event phenotypes with the PheCodes based on the ICD codes in the UK Biobank are publicly available for download at gate.genohub.org. The GWAS results are also available to browse on phewas.genohub.org, which contains manhattan plots, quantile-quantile plots, and locus zoom plots for each phenotype, as well as PheWAS plots for every genetic marker.

Human research participants

Policy information about studies involving human research participants and Sex and Gender in Research.

Reporting on sex and gender	Sex was used as a covariate to control for in the analysis model. Sex was determined based on the agreement of self-reporting and genetically determined (presence of Y chromosome) information.
Population characteristics	Participant in the UK Biobank studies (http://www.ukbiobank.ac.uk/), de-identified publicly available data.
Recruitment	UK Biobank recruited 500,000 people aged between 40-69 years in 2006-2010 from across the UK. More details can be found at http://www.ukbiobank.ac.uk/. FinnGen is a public-private partnership project combining genotype data from Finnish biobanks and digital health record data from Finnish health registries. Detailed description can be found in the manuscript, and also at https://www.finngen.fi/en.
Ethics oversight	UK Biobank data was obtained under the application number 52008. The UK Biobank EGF (https://www.ukbiobank.ac.uk/the-ethics-and-governance-council/) provides the required ethics oversight.<br>The Finngen study recruitment protocols followed the biobank protocols approved by Fimea. The Coordinating Ethics Committee of the Hospital District of Helsinki and Uusimaa (HUS) approved the FinnGen study protocol Nr HUS/990/2017. The FinnGen study is approved by Finnish Institute for Health and Welfare (THL), approval number THL/2031/6.02.00/2017, amendments THL/1101/5.05.00/2017, THL/341/6.02.00/2018, THL/2222/6.02.00/2018, THL/283/6.02.00/2019, THL/1721/5.05.00/2019, Digital and population data service agency VRK43431/2017-3, VRK/6909/2018-3, VRK/4415/2019-3 the Social Insurance Institution (KELA) KELA 58/522/2017, KELA 131/522/2018, KELA 70/522/2019, KELA 98/522/2019, and Statistics Finland TK-53-1041-17.

Note that full information on the approval of the study protocol must also be provided in the manuscript.

Field-specific reporting

Please select the one below that is the best fit for your research. If you are not sure, read the appropriate sections before making your selection.

☒ Life sciences ☐ Behavioural & social sciences ☐ Ecological, evolutionary & environmental sciences

For a reference copy of the document with all sections, see nature.com/documents/nr-reporting-summary-flat.pdf

Life sciences study design

All studies must disclose on these points even when the disclosure is negative.

Sample size	We analyzed publicly available UK Biobank data of white British samples (N=408,582), and data from the Finngen study release 5 (N=218,792). Since we analyzed a large number of phenotypes with a wide range of censoring rates, we didn't perform sample size calculation. We performed extensive type I error and power analysis using simulated data of sample size N=10,000 to compare the performance of different methods, and show that the proposed approach controls type I error and without losing power compared to the existing approaches.
Data exclusions	Non White British subjects were excluded from the analysis of the UK Biobank data. No subjects were excluded from the analysis of the Finngen study release 5 data.
Replication	We searched the GWAS catalog to check whether GWAS significant loci were known (replicated) or potentially novel. We also reported loci which are GWAS significant in the UK Biobank, and replicated in the Finngen study release 5 data. Experimental replication was not attempted as this is a methods paper, and novel discoveries are beyond the scope of the paper.
Randomization	Randomization of experimental groups was not required in this study. Covariates such as genetic ancestry principal components, sex, age were controlled for in the statistical analyses.
Blinding	We used de-identified coded data, and hence were blinded.

Reporting for specific materials, systems and methods

We require information from authors about some types of materials, experimental systems and methods used in many studies. Here, indicate whether each material, system or method listed is relevant to your study. If you are not sure if a list item applies to your research, read the appropriate section before selecting a response.

Materials & experimental systems

n/a	Involved in the study	
☒	☐	Antibodies
☒	☐	Eukaryotic cell lines
☒	☐	Palaeontology and archaeology
☒	☐	Animals and other organisms
☒	☐	Clinical data
☒	☐	Dual use research of concern

Methods

n/a	Involved in the study	
☒	☐	ChIP-seq
☒	☐	Flow cytometry
☒	☐	MRI-based neuroimaging
